# In vitro activity and resistance mechanisms of novel antimicrobial agents against metallo-β-lactamase producers

**DOI:** 10.1007/s10096-025-05080-1

**Published:** 2025-03-10

**Authors:** Matteo Boattini, Paolo Gaibani, Sara Comini, Cristina Costa, Rossana Cavallo, Francesco Broccolo, Gabriele Bianco

**Affiliations:** 1 Microbiology and Virology Unit, University Hospital Città Della Salute E Della Scienza Di Torino, Turin, Italy; 2 Department of Public Health and Paediatrics, University of Torino, Turin, Italy; 3 Lisbon Academic Medical Centre, Lisbon, Portugal; 4 Microbiology and Virology Unit, Department of Pathology, Azienda Ospedaliera Universitaria Integrata Di Verona, Verona, Italy; 5 Department of Diagnostic and Public Health, Microbiology Section, University of Verona, Verona, Italy; 6 Operative Unit of Clinical Pathology, Carlo Urbani Hospital, Ancona, Italy; 7 Department of Experimental Medicine, University of Salento, Lecce, Italy

**Keywords:** Metallo-β-lactamase, NDM, Avibactam, Taniborbactam, Zidebactam, Xeruborbactam, Cefiderocol, Durlobactam

## Abstract

The carbapenemase-producing Gram-negative organisms represent an urgent clinical and public health concern, as they have been associated with increased mortality and high dissemination in healthcare settings. Although overall incidence rates of infections sustained by metallo-β-lactamase (MβL)-producers have remained lower than those sustained by other carbapenemase-producers, albeit with substantial geographic differences, a significant increase in the prevalence of MβL-producers has been observed over the last decade. The recent development of new antimicrobials expanded the armamentarium to counter the challenge of metallo-β-lactamase (MβL)-producers. Cefiderocol and aztreonam/avibactam are already clinically available and recommended by international guidelines. In addition, two new classes of β-lactam/ β-lactamase combinations are under clinical evaluation: (*i*) combination of β-lactam with novel boronic-derived inhibitors (e.g. taniborbactam and xeruborbactam), (*ii*) combination of β-lactam with last generation diazabicyclooctane β-lactamase inhibitors (e.g. zidebactam and nacubactam), active on most of serine-β-lactamases but also showing strong intrinsic activity on PBP-2. This review aims to provide up-to-date data on the characteristics, activity and emerging resistance mechanisms of the armamentarium of clinically available or soon-to-be introduced drugs for the treatment of MβL-producing Gram-negative organisms.

## Introduction

The development and use of antibiotics since the second half of the twentieth century revolutionized the approach to the treatment and prevention of infectious diseases, enabling the evolution of modern medicine.

**Fig. 1:**
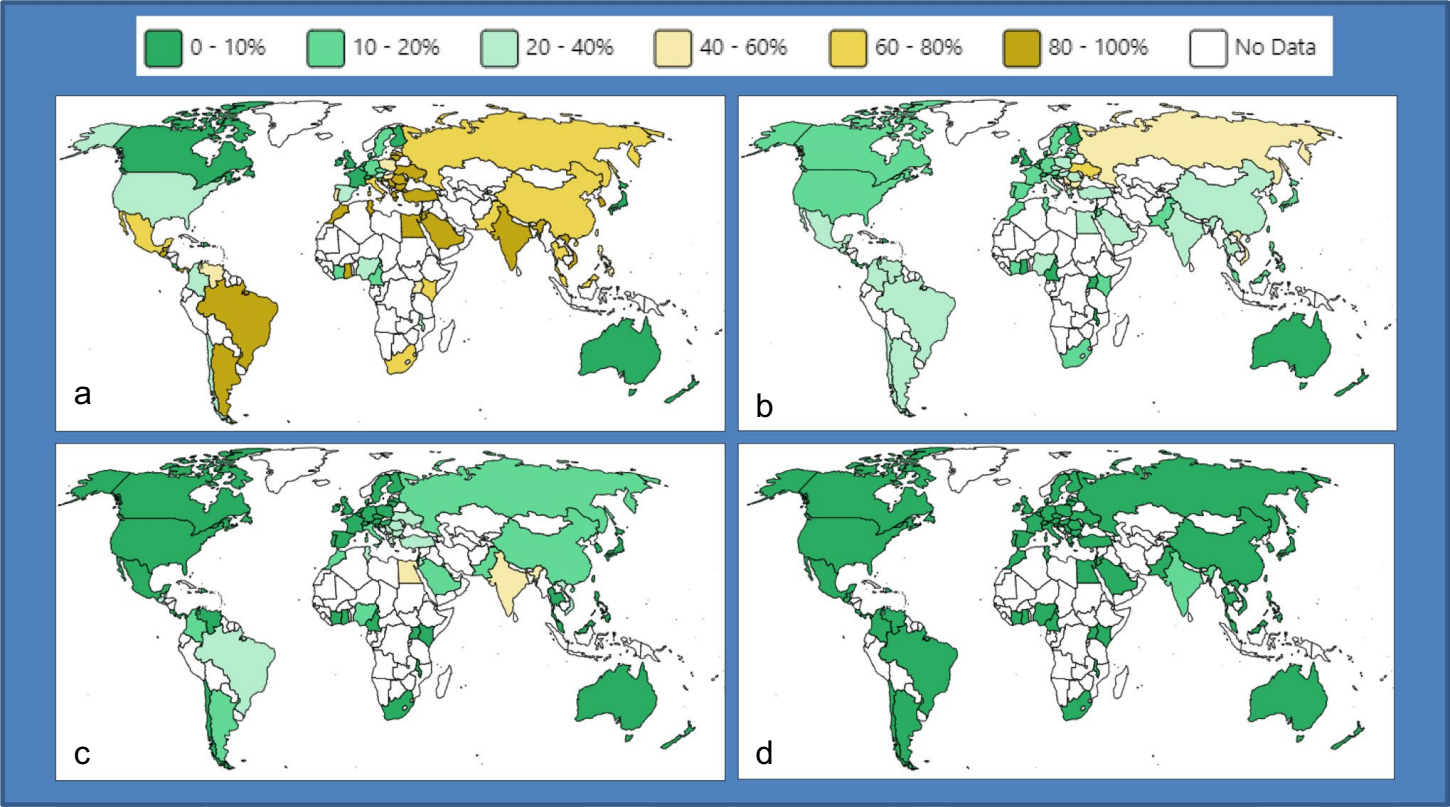
Global prevalence of carbapenem resistance (2013–2022) among clinical isolates of a. *Acinetobacter baumannii*, b. *Pseudomonas aeruginosa*, c. *Klebsiella pneumoniae*, d. *Escherichia coli*, according to the Antimicrobial Testing Leadership and Surveillance (ATLAS) database (available at: https://atlas-surveillance.com)

However, the huge increase in antimicrobial resistance (AMR) affecting all countries and healthcare sectors leads us to imagine a surreal scenario with a lack of access to effective antibiotic drugs in the near future. Bacterial AMR is estimated to have been directly responsible for 1.27 million global deaths and contributed to 4.95 million deaths in 2019, and the picture is expected to rise to 10 million per year by 2050 in the absence of effective interventions [1, 2]. The COVID-19 pandemic then exacerbated the concerns by accelerating the transmission and emergence of AMR [3–5]. Among the threats of AMR, carbapenems resistance is the most pressing, given the important role of this class of β-lactams in the clinical armamentarium [2]. The increase in the rate of carbapenem resistance, resulting in the global spread of carbapenem resistant organisms (CRO) (Fig. 1), was matched by an increase in associated deaths, from 619000 in 1990 to 1,03 million in 2021 [6]. Among CROs, Enterobacterales, *Pseudomonas aeruginosa*, and *Acinetobacter baumannii* are among the top three multi-drug-resistant pathogens on WHO’s priority list, being worthy of urgent study to develop new antibiotics (Fig. 1). The carbapenemase-producing subgroup of CROs is of great clinical and public health interest, as it has been associated with increased mortality and high dissemination in healthcare settings [7–9]. Several carbapenemases enzymes belonging to β-lactamases Ambler class A (e.g. *Klebsiella pneumoniae* carbapenemase, KPC), Ambler class B [metallo-β-lactamases, (MβLs)] and Ambler class D (oxacillinase, OXA-like) are largely reported to be associated with the global spread of CROs [7].

Although the overall incidence rates of infections sustained by MβL-producers have remained almost constant and lower than those sustained by other carbapenemase-producers (KPC- and OXA-like- producers), albeit with substantial geographic differences, a significant increase in the prevalence of MβL-producing CROs has been observed in recent years [1–7]. The recent introduction into clinical practice of new β-lactamase inhibitor combinations (e.g. ceftazidime/avibactam, meropenem/vaborbactam and imipenem/relebactam) may have contributed to this phenomenon, exerting strong selective pressure for the spread of MβLs, as the latter are not inhibited by the β-lactamase inhibitors approved to date [8].

Recently, cefiderocol and aztreonam/avibactam have been approved for the treatment of infections sustained by CRO, including MβL producers (Table 1). Moreover, new combinations of β-lactam/β-lactam inhibitors are under clinical evaluation and represent promising additional therapeutic options (Table 1).

**Table 1:** New antimicrobial agents, approved or under clinical investigation, with activity against metallo-β-lactamase producing Gram-negative bacilli

Antimicrobial agent	Characteristics	Year of FDA approval	Clinical trial	Inhibition profile				Direct activity of the β-lactamase inhibitor on PBPs	Targeted species	Resistance mechanisms
				SβLs			MβLs			
				A	C	D				
Cefiderocol	siderophore-cephalosporin	2019		NA	NA	NA	NA	NA	Enterobacterales, *P. aeruginosa*, *A. calcoaceticus-baumannii* complex, *S. maltophilia*	Mutations in genes related to iron transfer systems; alterations in PBP-3; expression of β-lactamases (mostly NDM-type) combined with other mechanism
Aztreonam/avibactam	monobactam + DBO inhibitor	-	Phase 3, NCT03580044	yes	yes	yes	no	no	Enterobacterales, *S. maltophilia, P. aeruginosa*	Mutation in PBP-3 encoding gene and concomitant expression of class C β-lactamases (e.g. CMY-45 and CMY-59)
Cefepime/taniborbactam	fourth-generation cephalosporin + cyclic boronate	-	Phase 3, NCT03840148; Phase 3, NCT06168734 (ongoing)	yes	yes	yes	yes	no	Enterobacterales, *P. aeruginosa*	IMP-like expression, NDM-9 or NDM-30 expression, alterations in PBP-3, loss of porins, upregulation of efflux pumps
Cefepime/zidebactam	fourth-generation cephalosporin + DBO inhibitor	-	Phase 3, NCT04979806	yes	yes	yes	no	Yes (PBP-2)	Enterobacterales, *P. aeruginosa*	Multiple mutations in genes encoding MexAB-OprM and its regulators, as well as PBP-2 and PBP-3; *bla*_PER-1_ overespression (*P. aeruginosa*)
β-lactam/xeruborbactam	β-lactam + cyclic boronate (ceftibuten/cefiderocol)	-	Ceftibuten/xeruborbactam: Phase 1, NCT06079775, NCT06157242 (ongoing) Cefiderocol/xeruborbactam: Phase 1, NCT06547554 (ongoing)	yes	yes	yes	yes	no	Enterobacterales, *P. aeruginosa, A. calcoaceticus-baumannii* complex	MexAB-OprM efflux pump overexpression (*P. aeruginosa*)
β-lactam/nacubactam	β-lactam (cefepime/aztreonam) + DBO inhibitor	-	Phase 3, NCT05887908 (completed) and NCT05905055 (ongoing)	yes	yes	yes	no	Yes (PBP-2)	Enterobacterales, *P. aeruginosa*	Mutations in PBP-2 encoding gene (*pbpA*); MexAB-OprM efflux pump overexpression, increased expression of PDC β-lactamase (*P. aeruginosa*)
Sulbactam/durlobactam	β-lactam derived by penicillin with β-lactamase inhibition activity (first generation) + DBO inhibitor	2023^a^		yes	yes	yes	no	Yes (PBP-2)	*A. baumannii*, Enterobacterales	MβL expression, alterations in PBP-3 and/or PBP-2 (*A. baumannii*)

^a^ Approved for treatment of hospital-acquired bacterial pneumonia and ventilator-associated bacterial pneumonia caused by susceptible strains of *Acinetobacter baumannii-calcoaceticus* complex

Abbreviations: FDA, food Drug Administration; PBP, penicillin binding protein; SβL, serine β-lactamase; MβL, metallo-β-lactamase; DBO, diazabicyclooctane; NA, not applicable

Herein, we reviewed current literature providing up-to-date data on *(i)* the epidemiological landscape of MβL-producing pathogens, *(ii)* the characteristics, activity and emerging resistance mechanisms of the latest clinically available or soon-to-be introduced drugs for treatment of MβL-producing Gram-negative infections.

## Metallo-β-lactamases

MβLs belong to Ambler class B, whereas class A, C and D include serine β-lactamases [10]. Serine β-lactamases essentially consist of two structural domains (an all α domain and an α/β domain) and the serine active-site is located in the groove between the two domains [11]. In MβLs (class B enzymes), the situation is more complex because the nucleophile is not one active-site serine, but an activated water/hydroxide coordinated to one or two Zn(II) ions, which in turn are coordinated by a set of amino acid ligands. The identity of these ligands falls into three patterns, which define the three subclasses of B-class enzymes, named B1, B2 and B3 [12].

Most of the MβLs identified so far belong to subclass B1, including the imipenemase (IMP), Verona imipenemase (VIM), and New Delhi MβL (NDM) families [13]. A limited number of enzymes belongs to subclass B2, including CphA and Sfh-I, produced by *Aeromonas* species (e.g. *A. hydrophila* and *A. veronii*) and *Serratia fonticola*, respectively. Subclass B3 includes around 50 distinct MβL enzymes, of which L1 MβL is clinically relevant being constitutively expressed by *Stenotrophomonas maltophilia*, an emerging multidrug-resistant Gram-negative organism causing healthcare acquired infections [14]. While the β-lactamase genes encoding class B2 and B3 enzymes have chromosomal localization, those of class B1 are largely plasmids borne and can readily spread by horizontal gene transfer both intra- and inter-species. Among more than 50 enzymes belonging to class B1, NDM, VIM and IMP MβLs are the most relevant from an epidemiological and clinical point of view. Their ability to hydrolyze all β-lactams except aztreonam, the lack of clinically usable inhibitors, their spread in several Gram-negative organisms such as Enterobacterales and non-fermenting species, as well as in nosocomial and environmental reservoirs make them one of the main and growing public health concerns [15]. In addition, new variants with higher affinity for zinc or requiring less of it are emerging, favoring their hydrolytic activity on β-lactam drugs in contexts of relative zinc scarcity, such as human infection sites [16, 17].

### IMP-type β-lactamases

The first MβL of the IMP group was identified in an imipenem-resistant *P. aeruginosa* clinical strain collected in 1988 in Japan [18]. The localization of the *bla*_IMP_ in a 47.7 kbp conjugative plasmid, pMS350, contributed to its spread to other bacterial species as it was subsequently found in the chromosome and as part of an integron in transferable plasmids of several clinical isolates of *P. aeruginosa*, *Pseudomonas putida*, *Pseudomonas stutzeri*, *Serratia marcescens* and *Citrobacter freundii* [19–23]. The first report of an IMP-type enzyme in Europe occurred in a MDR *A. baumannii* strain isolated from respiratory secretions of a critically ill patient in Italy [24]. Further analysis showed that the gene coded for IMP-2 and was carried in a gene cassette as part of a class I integron located on the chromosome. The *bla*_IMP-2_ gene cassette was located downstream of intI1 and the variable region also included aac(60)-Ib and ant(300)-Ia [24, 25]. The second identification of an IMP-type enzyme in Europe was carried out in Portugal in an *A. baumannii* strain isolated from urine [26]. Further analysis of that gene concluded that it was a new member, named *bla*_IMP-5,_ which followed the two new variants *bla*_IMP-3_ and *bla*_IMP-4_ previously identified in Asia [27, 28]. IMP-4 was subsequently found within a class 1 integron in isolates of *A. baumannii* and in similar integrons in strains of *A. pitti*, *K. pneumoniae*, *E. coli* and *Enterobacter cloacae*. [29, 30]. To date, the sequences of 102 variants of IMP MβLs, mostly identified in *P. aeruginosa*, *Enterobacter* spp., *K. pneumoniae* and *A. baumannii* clinical isolates are deposited in Genbank. IMP are still the predominant MβLs in Southeast Asia, where they are mostly detected in *P. aeruginosa, A. baumannii*, and Enterobacterales species. Considering *bla*_IMP_ variants in countries with high prevalence in Asia, *bla*_IMP−1_ was the most frequently reported in Japan (23%) and Singapore (50%). *bla*_IMP−4_ and *bla*_IMP−14_ were the most frequently reported in China (27%) and Thailand (27%), respectively [31]. However, recent regional or sporadic outbreaks have also been reported in the United States, Latin America (Brazil and Argentina), Australia, Lebanon, Egypt and some European countries such as Greece, France, United Kingdom, and Turkey [32–35].

### VIM-type β-lactamases

Among MβLs, VIM enzymes have cephalosporins as their preferred substrate and achieve a lower hydrolysis of carbapenem than that produced by enzymes of the IMP and NDM families [36].

The first two VIM variants, named VIM-1 and VIM-2, were identified in Italy and France in 1997 and 1996, respectively. They were both detetcted in *P. aeruginosa* isolates containing *bla*_VIM_ gene cassettes inserted into a class 1 integron [37, 38]. Despite the high amino acid sequence identity, the two genes had a different location: *bla*_VIM-1_ was located within the chromosome and included a second aac(60)-Ib-containing gene cassette, whereas *bla*_VIM-2_ was included in a unique gene cassette, located in an integron within a ~ 45-kbp non-conjugative plasmid. Furthermore, the enzymes were not closely related to other MβL, with only 28–31% sequence identity between VIM-1/VIM-2 and IMP-1 [37, 38]. After the first identification, VIM enzymes spread rapidly throughout Southern Europe, with outbreaks in Italy and Greece in 2006, first in isolates of *P. aeruginosa* and then of *K. pneumoniae* [33, 39–41]. Until 2017, VIM-type was the predominant MβLs in Europe, especially in Mediterranean countries.

The rapid global spread of VIM MβLs, especially in *Enterobacteriacea*e and *Pseudomonas*, has led to the identification of a large number of new variants in the recent years (87 uploaded on Genbank, last accessed on September 2024). Currently, VIM MβLs are found globally, mainly in *K. pneumoniae*, *E. cloacae* complex and *P. aeruginosa* [33]. VIM-2-like MβLs are mostly reported in *P. aeruginosa*, whereas VIM-1-like MβLs (e.g. VIM-4) are frequently reported in *Enterobacteriaceae* species. Furthermore, the presence of VIM variants (VIM-1, VIM.2, VIM-3, VIM-6, VIM-11, VIM-25) in *A. baumannii* isolates has been reported in Korea, Greece, Saudi Arabia and Iran since the early 2000s [42].

### NDM-type β-lactamases

The NDM MβL was first described in 2009 in a *K. pneumoniae* isolate from a urine sample of a Swedish patient, previously admitted to two Indian hospitals [43]. The *bla*_NDM-1_ was located in a 180 kbp plasmid including multiple antibiotic resistance genes. BLAST analysis showed that NDM-1 shared very little sequence homology with other MβLs, and the closest relative was VIM-1, with only 32.4% of amino acid identity [44]. The detection of a MDR *E. coli* strain harboring the same *bla*_NDM-1_-carrying plasmid suggested that plasmid transfer by conjugation occurred with high frequency, and this was then demonstrated by in vitro conjugation assays [43]. The rapid spread of *bla*_NDM-1_-carrying plasmid in many species of enteric bacteria, foodborne pathogens (*Shigella* spp., *Vibrio cholerae*), and non-fermenting Gram-negative species (*A. baumannii*, *P. aeruginosa*) led to its worldwide dissemination [33, 41, 45, 46]. According to Genbank data (last accessed on September 2024), 68 different variants of *bla*_NDM_ were identified to date. In addition to multiple sequences of the gene, several plasmids carrying *bla*_NDM-like_ and different sequence typing of the species involved were identified, demonstrating the promiscuity of *bla*_NDM_.

Currently, *bla*_NDM_ is endemic not only in the Indian subcontinent but also in the Asia–Pacific region, Balkan countries, Eastern Europe, North Africa and Arabian Peninsula [33, 46–48]. Furthermore, regional or sporadic health-care dissemination of NDM-producing *Enterobacteriaceae* in Latin America, USA, and many Western European countries such as, Netherlands, Denmark, Spain, and Italy was recently reported [49–55]. A recent surveillance study involving 24,580 carbapenem-resistant Enterobacterales isolates collected in 2020–2022 from 64 medical centers located in Europe, Latin America, and Asia–Pacific region showed that NDM was the second most common carbapenemase (29.9%) after KPC (44.6%). Its occurrence was highest in the Asia–Pacific region (55.4%), followed by Latin America (31.7%), Eastern Europe (27.3%) and Western Europe (15.7%) [56]. Similar finding emerged by a surveillance study involving Enterobacterales isolates (n = 34.623) collected in 86 US hospitals from 2016 to 2020 [57]. Among MBL-positive isolates globally collected during the period 2016–2020, NDM-positive was the most common genotype collected globally (83.3%); NDM-1, NDM-5 and NDM-7 were the most prevalent variants (61.4%, 32.4% and 4.2%, respectively) followed by NDM-4, NDM6, NDM-9, NDM-16, NDM-19, NDM-24 (overall 2%) [58].

## Therapeutic options for MβLs

In addition to inactivation by metal chelators, all MβLs share further functional characteristics, including hydrolytic activity on carbapenems, resistance to the clinically available β-lactamase inhibitors (e.g. clavulanate, sulbactam, tazobactam, avibactam, vaborbactam, relebactam) and no activity against monobactams. Moreover, the location of MβL encoding genes in genomic contexts with multiple resistance determinants is often associated to resistance towards more drug classes other than β-lactams. As a result, the optimization of antibiotic therapy of infections sustained by MβL-producers is challenging. Although “old” drugs such colistin, fosfomycin, tetracyclines and aminoglycosides may show in vitro efficacy, they are associated with less bactericidal activity or more toxicity [48]. Recently, cefiderocol and aztreonam/avibactam have been approved by the FDA and/or EMA agencies for treating MβL-producing pathogens infections.

**Fig. 2:**
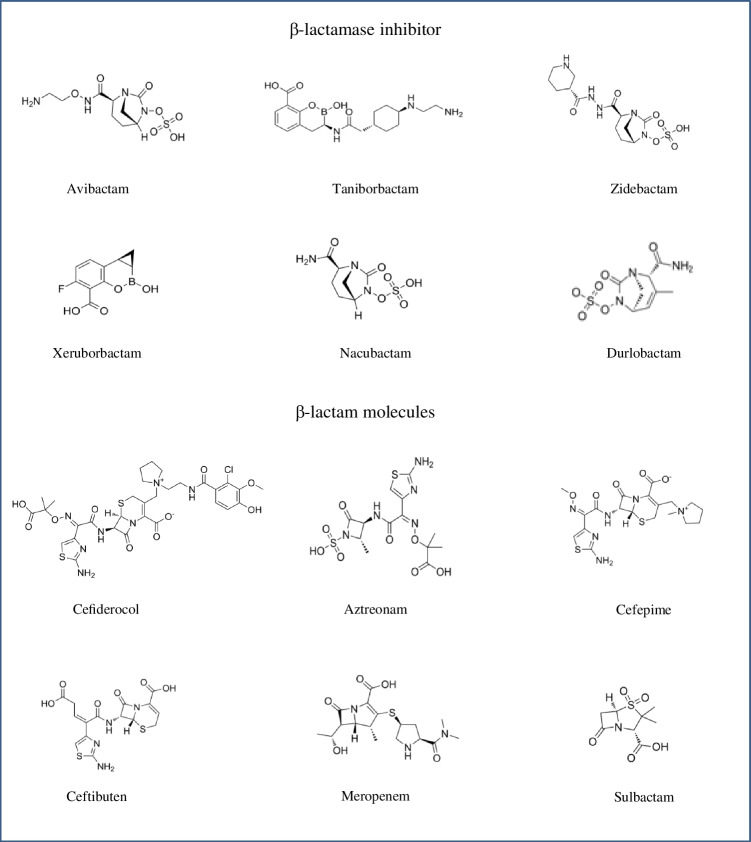
Chemical structures of β-lactamase inhibitors and β-lactams analyzed in this review

Moreover, recent research has been increasingly focused on broad spectrum β-lactamase inhibitors. Bicyclic boronates have been developed as successful inhibitors of both MBLs and serine β-lactamases. Two bicyclic boronates, taniborbactam and xeruborbactam, were proposed as promising candidates for dual inhibitors of MBLs and serine β-lactamases **(**Fig. 2 and Table 1**)**. Moreover, the development of new non-β-lactam antibiotics targeting penicillin binding proteins (PBPs) is another option taken in consideration to counter MBL-mediated resistance. For instance, the diazabicyclooctane derivative zidebactam, nacubactam and durlobactam shows activity against PBP-2, as well as inhibition of the main serine β-lactamases (Fig. 2 and Table 1).

### Cefiderocol

Cefiderocol (formerly S-649266, GSK2696266) is a new siderophore cephalosporin developed and marketed by Shionogi & Co., Ltd. as a promising drug for the treatment of multidrug-resistant Gram-negative bacilli infections (Fig. 2 and Table 1). It was approved by FDA and EMA on November 2019 and April 2020, respectively.

The unique characteristic of binding to extracellular free iron via a siderophore side chain allows active transport into the periplasmic space of Gram-negative bacteria via active iron transport systems. Therefore, unlike other β-lactams, cefiderocol uses both this active iron transport and the traditional porin-mediated transport system to enter the bacterial cell and target PBPs. This ‘Trojan horse’ strategy of action allows cefiderocol to overcome the resistance mechanisms that alter permeability of the outer membrane (e.g. overexpression of efflux pumps, loss of the porin channels) [59]. Moreover, its structure, similar to that of cefepime and ceftazidime but with the addition of different constituent groups, confers an enhanced stability to the action of β-lactamases including MβL [59].

In vitro activity of cefiderocol against carbapenem-non-susceptible and MβL-producing pathogens was investigated in several studies, including surveillance reports and a recent meta-analysis (Table 2) [60–77]. Using the EUCAST/CLSI breakpoint thresholds, cefiderocol susceptibility rates were generally high in carbapenem-non-susceptible pathogens (82.5–92.6%, 94.8–98.5%, 88.6–91.8% in Enterobacterales, *P. aeruginosa* and *A. baumannii* complex isolates, respectively), lower in MβL producers (72.1–86.6%, 94.3–97.5%, 51.4–75.8% in Enterobacterales, *P. aeruginosa* and *A. baumannii* complex isolates, respectively), and even lower in NDM-producing isolates (50.5–75%, 71.2–82.1%, 47.6–71.5%, in Enterobacterales, *P. aeruginosa* and *A. baumannii* complex isolates, respectively) (Table 2). As shown, there were significant differences in cefiderocol susceptibility rates when comparing results between EUCAST and CLSI breakpoints, which was not the case with *S. maltophilia* (97.2–99.2%).

**Table 2:** In vitro activity of cefiderocol against MDR Gram-negative clinical isolates collections including metallo-β-lactamase producers

Reference	Country	Period of isolates collection	Breakpoint	Carbapenem non-susceptible			MβL producers				NDM producers		
				Enterobacterales	*P. aeruginosa*	*ACB*	Enterobacterales	*P. aeruginosa*	*ACB*	*S. maltophilia*	Enterobacterales	*P. aeruginosa*	*ACB*
[60]	Worldwide	2006–2023	CLSI	6638/7175; 92.5%	4321/4389; 98.4%	5560/6047; 91.9%	1400/1679; 83.4%	540/562; 96.1%	74/93; 79.5%	2922/3003; 97.3%	1096/1476; 74.2%	33/41; 80.5%	66/85; 77.6%
			EUCAST	4614/5589; 82.5%	3823/4041; 96.4%	4296/4831; 88.9%	1064/1507; 70.6%	495/527; 93.9%	55/93; 59.1%	3019/3030; 99.6%	490/1024; 47.8%	37/51; 72.5%	47/85; 55.3%
[61]	China	2014–2022	CLSI	289/320; 90.3%	-	-	49/57; 86%	-	-	-	49/57; 86%	-	-
[62]	Worldwide	2019–2021	CLSI	-	790/806; 98%	-	-	160/164; 97.5%	-	-	-	11/13; 84.6%	-
			EUCAST	-	766/806; 95%	-	-	147/164; 89.6%	-	-	-	9/13; 69.2%	-
[63]	Swiss	2022–2023	EUCAST	-	31/39; 79.5%		31/39; 79.5%				6/12; 50%		
[64]	Japan	2019–2020	CLSI	300/307; 97.7%	18/18; 100%	49/57; 86%	272/278; 97.8%	16/16; 100%	8/10; 80%	-	20/24; 83.3%	-	1/3; 33.3%
[65]	Italy	2019–2021	EUCAST	108/124; 87.1%	25/26; 96.1%	68/70; 97.1%	8/12; 66.7%	3/4; 75%	5/6; 83.3%	12/12; 100%	0/2; 0%	-	5/6; 83.3%
[66]	Spain	2015–2020	EUCAST	83/90; 92.2%	-	-	28/35; 80%	-	-	-	8/14; 57.1%	-	-
[67]	North America and Europe	2014–2019	CLSI	181/198; 91.4%	227/227; 100%	15/25; 60%	181/198; 91.4%	227/227; 100%	15/25; 60%	-	80/94; 85.1%	2/2; 100%	11/21; 52.4%
			EUCAST	133/198; 67.2%	221/227; 97.3%	11/25; 44%	133/198; 67.2%	221/227; 97.3%	11/25; 44%	-	49/94; 52.1%	1/2; 50%	7/21; 33.3%
[68]	Spain	2015-2020	CLSI	153/160; 95.6%	68/68; 100%	-	153/160; 95.6%	68/68; 100%	-	-	-	-	-
			EUCAST	129/160; 80.6%	68/68; 100%	1/4; 25%	129/160; 80.6%	68/68; 100%	1/4; 25%	-	-	-	-
[69]	Taiwan	2013–2021	CLSI	171/195; 87.7%	-	-	123/143; 86%	-	-	-	58/74; 78.4%	-	-
[70]	Türkiye	2017	EUCAST	-	233/244; 95.5%	-	-	13/14; 92.8%	-	-	-	4/5; 80%	-
[71]	Europe	2020	EUCAST	130/148; 87.8%	-	-	20/35; 57.1%	-	-	-	13/27; 48.1%	-	-
			CLSI	139/148; 93.9%	-	-	26/35; 74.2%	-	-	-	19/27; 70.4%	-	-
[72]	Poland	2019–2022	EUCAST	60/60; 100%	-	-	60/60; 100%	-	-	-	60/60; 100%	-	-
[73]	Mexico	2012–2022	CLSI	-	-	-	-	-	-	96/101; 95%	-	-	-
[74]	Europe	2020	EUCAST	-	135/139; 97.1%	193/227; 85%	-	29/30; 96.7%	0/12; 0%	-	-	1/2; 50%	0/12; 0%
[75]	Northern Ireland, Spain and the Netherlands	-	EUCAST	-	-	-	-	-	-	88/102; 86.3%	-	-	-
[76]	Taiwan	2019–2021	CLSI	-	110/110; 100%	122/129; 94.6%	-	-	-	46/47; 97.9%	-	-	-
[77]	Italy	2019–2020	EUCAST	31/41; 75.6%	7/8; 87.5%	-	31/41; 75.6%	7/8; 87.5%	-	-	1/9; 11.1%	-	-
Pooled data		2006–2023	CLSI	7871/8503; 92.6%	5534/5618; 98.5%	5746/6258; 91.8%	2006/2315; 86.6%	1011/1037; 97.5%	97/128; 75.8%	3064/3151; 97.2%	1196/1594; 75%	46/56; 82.1%	78/109; 71.5%
			EUCAST	5288/6410; 82.5%	5309/5598; 94.8%	4569/5157; 88.6%	1504/2087; 72.1%	983/1042; 94.3%	72/140; 51.4%	3119/3144; 99.2%	627/1242; 50.5%	52/73; 71.2%	59/124; 47.6%

Susceptibility data were re-interpreted according to:

EUCAST susceptibility breakpoint (v_14.0, 2024): ≤ 2 mg/L;

CLSI susceptibility breakpoints (CLSI M100 ED34:2024): Enterobacterales, *Pseudomonas*, *Acinetobacter*, ≤ 4 mg/L; *S. maltophilia* ≤ 1 mg/L

Abbreviation: *ACB*, *Acinetobacter baumannii-calcoaceticus* complex

In vivo activity of cefiderocol against MβL-producing pathogens was evaluated in the CREDIBLE-CR and APEKS-NP studies [78–80]. Overall, cefiderocol monotherapy was effective in the treatment of infections sustained by MβL-producing Gram-negative bacteria. The rates of clinical cure (70.8%), microbiological eradication (58.3%) and all-cause mortality at 28 days (12.5%) compared favorably with the best available therapy and high-dose meropenem (40.0%; 30.0%; and 50.0%), respectively. Clinical recovery was lower for NDM-producing infections (56.2%) than for non-NDM-producing infections (100%) [78–80].

In vivo emergence of cefiderocol resistance following therapy with cefiderocol or other β-lactams (e.g. ceftazidime/avibactam and ceftolozane/tazobactam) against *P. aeruginosa*, *A. baumannii* complex and Enterobacterales infections was reported [81–86]. Resistance to cefiderocol was shown to be a consequence of combinations of various mechanisms, including mutations in genes related to iron transfer systems (e.g. *piuA*, *pirA, cirA* and *tonB*), expression of β-lactamases (e.g. NDM-type, KPC variants linked to ceftazidime/avibactam resistance, OXA-427, CMY-185, CMY-186 and PER-type), mutations in penicillin binding protein PBP-3, porin loss and efflux pump overexpression [59].

### Aztreonam/avibactam

Aztreonam/avibactam (Emblaveo, Pfizer) is a combination including a monobactam that interferes with bacterial cell wall synthesis and a non-β-lactam β-lactamase inhibitor that is active against class A, class C and some class D β-lactamases (Fig. 2 and Table 1). It was approved by the EMA on April 2024 for patients suffering from MDR infections and limited treatment options, including complicated intra-abdominal infections (cIAI), hospital-acquired pneumonia (HAP), and complicated urinary tract infections (cUTI) [87]. Although aztreonam is not hydrolyzed by MβLs, co-expression of MβLs with β-lactamases of the other Ambler classes able to hydrolyze aztreonam is frequent. Therefore, aztreonam monotherapy is often not active against MβL-producing strains. Pending regulatory agencies approval, co-administration of ceftazidime/avibactam and aztreonam has been recommended for the treatment of MβL-producing Enterobacterales infections by both the Infectious Disease Society of America (IDSA) and the European Society of Clinical Microbiology and Infectious Diseases (ESCMID) [88, 89].

Several studies have evaluated the in vitro activity of aztreonam/avibactam against worldwide isolates of Enterobacterales and *P. aeruginosa* exhibiting carbapenem non-susceptibility and/or MβL-production [48, 53, 56, 58, 63, 69, 71, 74, 76, 90–97] (Table 3). According to EUCAST/CLSI 2024 breakpoints, among the 88,592 carbapenem-non-susceptible and/or carbapenemase-producing Enterobacterales isolates tested, the pooled susceptibility rate was 99.5%, and only a small reduction was observed in the MβL- or NDM- producers subgroups (96.9% and 95.6%, respectively). Moreover, a recent report showed excellent in vitro activity of aztreonam/avibactam against Enterobacterales isolates producing dual-carbapenemase (MβL + class A carbapenemase, n = 14; MβL + class D carbapenemase, n = 35), revealing 100% susceptibility and overall MIC_50_ and MIC_90_ of ≤ 0.25 mg/L and 0.5 mg/L, respectively [98].

**Table 3:** In vitro activity of aztreonam/avibactam against MDR Gram-negative clinical isolates collections including metallo-β-lactamase producers

Reference	Country	Period of isolates collection	Breakpoint	Carbapenem non-susceptible		MβL producers		NDM producers	
				Enterobacterales	*P. aeruginosa*	Enterobacterales	*P. aeruginosa*	Enterobacterales	*P. aeruginosa*
[48]	China	2021	CLSI	298/306; 97.4%	71/138; 51.4%	99/102; 97%	8/15; 53.3%	-	-
			EUCAST	298/306; 97.4%	97/138; 70.3%	99/102; 97%	11/15; 73.3%	-	-
[63]	Swiss	2022–23	EUCAST	-	34/39; 87.2%	-	34/39; 87.2%	-	-
			CLSI	-	16/39; 41%	-	16/39; 41%	-	-
[53]	USA	2019–21	CLSI/EUCAST	258/261; 98.8%	-	32/33; 97%	-	28/29; 96.5%	-
[56]	Worldwide	2020–22	CLSI/EUCAST	1011/1016; 99.5%	-	356/356; 100%	-	-	-
[58]	Worldwide	2016–2020	CLSI/EUCAST	82,642/82.785; 99.8%	-	1681/1707; 98.5%	-	1395/1421; 98.2%	-
[69]	Taiwan	2013–2021	CLSI/EUCAST	189/195; 96.9%	-	137/143; 95.8%	-	69/74; 93.2%	-
[71]	Europe	2020	EUCAST/CLSI	140/148; 94.6%	-	35/35; 100%	-	27/27; 100%	-
[74]	Europe	2020	EUCAST	-	58/139; 41.7%	-	22/30; 73.3%	-	1/2, 50%
			CLSI	-	17/139; 12.2%	-	9/30; 30%		1/2, 50%
[76]	Taiwan	2019–2021	CLSI	-	14/110; 12.7%	-	-	-	-
			EUCAST	-	44/110; 40%	-	-	-	-
[90]	Spain	2018	CLSI/EUCAST	54/55; 98.2%	-	54/55; 98.2%	-	9/10; 90%	-
[91]	China	2019	CLSI/EUCAST	110/119; 92.4%	-	35/44; 79.5%	-	32/41; 78%	
[92]	UK	2015, 2017, 2019	CLSI/EUCAST	413/464; 89%	-	413/464; 89%	-	193/243; 79.4%	-
[93]	Europe	2019–2020	CLSI/EUCAST	421/424; 99.3%	-	109/109; 100%	-	81/81; 100%	-
[94]	Worldwide	2016–2017	CLSI/EUCAST	582/583; 99.8%	-	114/114; 100%	-	-	-
[95]	China	2016–2017	CLSI/EUCAST	161/161; 100%	-	161/161; 100%	-	151/151; 100%	-
[96]	Worldwide	2012–2015	EUCAST	1378/1498; 92%	-	249/267; 93.2%	319/452; 70.6%	-	-
			CLSI	1378/1498; 92%	8692/11842; 73.4%	249/267; 93.2%	280/452; 61.9%	-	-
[97]	Worldwide	2012–2013	EUCAST	537/577; 93.1%	3246/3766; 86.2%	91/91; 100%	88/118; 74.6%	-	-
			CLSI	537/577; 93.1%	2772/3766; 73.6%	91/91; 100%	52/118; 44.1%	-	-
Pooled data		2012–2023	EUCAST	88,196/88592; 99.5%	3479/4192; 83%	3566/3681; 96.9%	474/654; 72.5%	1985/2077; 95.6%	1/2; 50%
			CLSI	88,196/88592; 99.5%	11,582/16034; 72.2%	3566/3681; 96.9%	365/654; 55.8%	1985/2077; 95.6%	1/2; 50%

For susceptibility testing purpose, the concentration of taniborbactam was fixed at 4 mg/L

No available clinical breakpoints for aztreonam/avibactam. Susceptibility data were re-interpreted according to aztreonam susceptibility breakpoints as follows:

EUCAST susceptibility breakpoint (v_14.0, 2024): Enterobacterales, ≤ 4 mg/L; *Pseudomonas*, ≤ 16 mg/L

CLSI susceptibility breakpoints (CLSI M100 ED34:2024): Enterobacterales, ≤ 4 mg/L; *Pseudomonas*, ≤ 8 mg/L

Conversely, lower rates of aztreonam/avibactam susceptibility were reported among carbapenem-non-susceptible (72.2–83%) and MβL-producing *P. aeruginosa* isolates (55.8–72.5%). These findings were consistent with data on MIC_50_ and MIC_90_ (0.125 mg/L to 0.25 mg/L *vs.* 16 to 32 mg/L, in Enterobacterales and *P. aeruginosa*, respectively) [99]. This difference in susceptibility could be due to the presence of multiple resistance mechanisms commonly detected in *P. aeruginosa*, such as overexpression of efflux systems, production of PDC-like, PER-like and OXA-like β-lactamase variants, and loss of porins. Consequently, these data might suggest the use of aztreonam/avibactam mainly for the treatment of infections sustained by MβL-producing Enterobacterales [99]. Aztreonam-avibactam showed also to be a promising β-lactam/β-lactamase-inhibitor combination against MDR *S. maltophilia* [100, 101]. Sader et al. evaluated the in vitro activity of aztreonam/avibactam against 1.839 *S. maltophilia* isolates collected worldwide and showed high activity, regardless of the geographic region or type of infection (overall MIC_50/90_, 4/4 mg/L; 97.8% inhibited at ≤ 8 mg/L [101].

As far as in vivo studies are concerned, a phase 2a trial showed both relevant attainment of PK/PD targets and favorable benefit–risk ratio for aztreonam/avibactam [102].The recommended daily dose for aztreonam/avibactam was a 30-min infusion with 500/167 mg aztreonam/avibactam as loading dose and maintenance dose with 3-h infusions of 1500/500 mg aztreonam/avibactam every 6 h. This resulted in a higher daily dose of avibactam as compared to the combination aztreonam plus ceftazidime/avibactam dosing (2-h infusion of ceftazidime/avibactam, 2000/500 mg every 8 h with aztreonam, 2000 mg every 6 h) [102, 103]. The REVISIT phase 3 trial (NCT03329092; registration date: 2017–10-06; https://clinicaltrials.gov/study/NCT03329092) assessed aztreonam/avibactam ± metronidazole compared to meropenem ± colistin in patients suffering from cIAI and HAP/VAP caused or suspected to be caused by Gram-negative bacteria. The cure rate of patients with cIAI and treated with aztreonam/avibactam was higher than that of those treated with meropenem (85.1% *vs.* 79.5%). In cases of patients with HAP, the aztreonam/avibactam cure rate was lower (46.7% *vs*. 54.5%). The 28-day mortality rates were low for both groups (1.9% and 2.9% for the aztreonam/avibactam and the meropenem group, respectively) [103, 104]. The ASSEMBLE phase 3 trial was early terminated due to difficulty in recruiting patients. However, before termination, 5/12 (41.7%) patients with confirmed MβL Gram-negative infections were cured with aztreonam/avibactam and none out three with best-available therapy (NCT03580044; registration date: 2018–06-04; https://clinicaltrials.gov/study/NCT03580044).

In vivo emergence of resistance to aztreonam/avibactam has been unfrequently reported in the real-world experience [105, 106]. Mutations in genes encoding for PBP-3 (*ftsl*) and expression of mutated AmpC β-lactamase CMY were identified as potential resistance mechanisms occurred in NDM-5-producing *E. coli* following aztreonam plus avibactam based-therapies [105, 106]. Resistance to aztreonam/avibactam is increasingly reported in *E. coli* in Asia [107, 108] and Europe [109–111] due to co-expression of PBP-3 mutations and NDM. Moreover, since PBP-3 is also a target of other β-lactams, occurrence of co-resistance to cefiderocol was reported [105, 106]. The most commonly reported aztreonam/avibactam non-susceptible clones at high-risk are those carrying mutations in PBP-3, in particular a four amino acid insertion (YRIN/K) at residue 333 or 338 of PBP-3 [105, 106, 112–117]. However, presence of mutated PBP-3 alone may not be sufficient to confer high-level resistance, and concomitant production of class C β-lactamases (e.g. CMY-45 and CMY-59) was often observed [105, 106, 112, 114–117]. Although resistance to aztreonam/avibactam was essentially observed in high-risk clones of *E. coli*, co-resistance to ceftazidime/avibactam and aztreonam/avibactam in *K. pneumoniae* was correlated with expression of mutated KPC enzymes [86, 118].

### Cefepime/taniborbactam

Taniborbactam (formerly VNRX-5133, Venatorx Pharmaceuticals) belongs to the cyclic boronate family and exhibits β-lactamase inhibitory activity against KPC, OXA-48 and some MβLs (VIM and NDM but not IMP) (Fig. 2 and Table 1) [119–121]. This compound was the first boronate inhibitor to show direct inhibitory activity against serine β-lactamases and MβL enzymes via different mechanisms. While avibactam is exclusively an inhibitor of serine β-lactamases, the addition of an aromatic group with a carboxylic acid to the boronate ring confers taniborbactam the ability to bind MβL enzymes as well [122]. In steady-state kinetic analysis experiments, taniborbactam was confirmed as a competitive inhibitor of VIM-2 and NDM-1 but not IMP-1 [inhibition constant (Ki) of 0.019, 0.081 μM and 30 μM, respectively] [123]. Moreover, inhibitory activity of taniborbactam was shown against various class A and C enzymes and OXA-48 like class D, with Ki values similar to those of avibactam. Taniborbactam inhibits serine β-lactamases through slow dissociation, while also acting as a reversible competitive inhibitor with a low Ki and rapid dissociation from MβLs [123].

A global surveillance study assessed *in vitro* activity of cefepime/taniborbactam against a 2018–2020 worldwide collection of Enterobacterales (*n* = 13,731) and *P. aeruginosa* (*n* = 4,619) isolates [124]. Using the fixed concentration 4 mg/L of taniborbactam, the MIC_50_/MIC_90_ were 0.06/0.25 mg/L, 2/8 mg/L*,* and rates of inhibition at ≤ 16 μg/mL or ≤ 8 μg/mL were 99.7%/99.5% and 97.4%/94.2% in Enterobacterales and *P. aeruginosa*, respectively [124]. Data on in vitro activity of cefepime/taniborbactam against carbapenem-non-susceptible and/or carbapenemase-producers, and MβL-positive Enterobacterales and *Pseudomonas* spp. was reported in Table 4. According to the proposed provisional susceptibility breakpoint (≤ 16 mg/L) [124], the pooled susceptibility rates were 86.7% and 82% for Enterobacterales and *Pseudomonas* spp, respectively, followed by 72.3% and 77.3% in the respective MβL-positive subgroups. Among MβL-positive isolates, in vitro activity was higher among VIM-positive than NDM-positive isolates (98.7% *vs.* 64.1% in Enterobacterales, and 81.4% *vs.* 0% in *Pseudomonas* spp, respectively). Interpretation of the overall MIC values using the susceptibility breakpoints of cefepime from EUCAST (2024) and CLSI (2024) led to a significant reduction in susceptibility rates with values below 60% in the overall MβL-positive isolates (range 47–58.3%) and below 50% in the NDM-positive Enterobacterales (ramge 36.4–43.4%) (Table 4). Of note, various studies showed a considerable discrepancy in susceptibility rates to cefepime/taniborbactam [63, 71, 74, 124–130]. For instance, among NDM-positive Enterobacterales, susceptibility rates (≤ 16 mg/L) of 90–100% were reported in Spain [128, 129], 96.3% in Europe [71], 86.5% in a worldwide collection [124], 79.9% in the UK [126], 66.7% in China [125] and 28% in India [130]. These differences in data could be due to the different geographical distribution of bacterial clones harboring resistance mechanisms such as the expression of specific β-lactamase variants [131–133]. Genomic characterization of cefepime/taniborbactam-resistant Enterobacterales strains showed that multiple mechanisms may be associated with cefepime/taniborbactam resistance, including production of IMP-like carbapenemases, alterations in PBP-3, loss of porins (OmpA, OmpR, Omp35, OmpK36), upregulation of efflux pumps, often with concomitant expression of NDM variants or class D β-lactamases [124, 129, 131–133]. Terrier et al. showed that that taniborbactam exhibits an overall excellent activity against B1 MβLs including most NDM- and VIM-like as well as SPM-1, GIM-1, and DIM-1 enzymes, but not against NDM-9, NDM-30 (differing from NDM-1 by a single amino acid substitution), and VIM-1 like enzymes (particularly VIM-83) [134, 135]. Furthermore, Drusin et al. revealed that the replacement of Glu149 by a Lys residue in NDM-9 results in a reduction of taniborbactam affinity and activity [136]. Similarly, WGS characterizations have identified multiple resistance mechanisms in *P. aeruginosa* isolates displaying high MICs of cefepime/taniborbactam, such as IMP production, PBP-3 mutations, upregulation of efflux pumps, and overexpression of AmpC beta-lactamase (PDC) [124, 129].

**Table 4:** In vitro activity of cefepime/taniborbactam against MDR Gram-negative clinical isolates collections including metallo-β-lactamase producers

Reference	Country	Period of isolates collection	Breakpoint	Carbapenem non-susceptible and/or carbapenemase-producers		MβL producers		NDM producers		VIM producers		IMP producers
				Enterobacterales	*P. aeruginosa*	Enterobacterales	*P. aeruginosa*	Enterobacterales	*P. aeruginosa*	Enterobacterales	*P. aeruginosa*	Enterobacterales
[63]	Swiss	2022–23	EUCAST/CLSI	-	20/39; 51.3%	-	20/39; 51.3%	-	-	-	-	-
			Provisional BP	-	21/39	-	21/39	-	-	-	-	-
[71, 74]	Europe	2020	EUCAST	139/145	83/139; 59.7%	24/37; 64.9%	16/30; 53.3%	19/27; 70.4%	0/2; 0%	5/7; 71.4%	15/24; 62.5%	-
			CLSI	124/145; 85.5%	83/139; 59.7%	18/34; 52.9%	16/30; 53.3%	13/27; 48.1%	0/2; 0%	5/7; 71.4%	15/24; 62.5%	-
			Provisional BP	144/145	114/139; 82%	33/34; 97%	18/30; 60%	26/27; 96.3%96.3%	0/2; 0%	7/7; 100%	17/24; 70.8%	-
[123]	-	2005–2018	EUCAST	59/60; 98.3%	38/41; 92.7%	19/20; 95%	5/5; 100%	9/9; 100%	-	8/8; 100%	5/5; 100%	-
			CLSI	57/60; 95%	38/41; 92.7%	17/20; 85%	5/5; 100%	8/9; 88.9%	-	7/8; 87.5%	5/5; 100%	-
			Provisional BP	60/60; 100%	39/41; 95.1%	20/20; 100%	5/5; 100%	9/9; 100%	-	8/8; 100%	5/5; 100%	-
[124]	Worldwide	2018–2020	EUCAST	534/625; 85.4%	151/216; 69.9%	158/229; 69%	120/159; 75.5%	139/207; 67.1%	-	19/22; 86.4%	120/159; 75.5%	-
			CLSI	472/625; 75.5%	151/216; 69.9%	150/229; 65.5%	120/159; 75.5%	132/207; 63.8%	-	18/22; 81.8%	120/159; 75.5%	-
			Provisional BP	595/625	177/216; 81.9%	201/229; 87.8%	139/159; 87.4%	179/207; 86.5%	-	22/22; 100%	139/159; 87.4%	-
[125]	China	2017–2019	EUCAST	132/207; 63.8%	15/21; 71.4%	37/87; 42.5%	-	37/87; 42.5%	-	-	-	-
			CLSI	105/207; 50.7%	15/21; 71.4%	30/87; 34.5%	-	30/87; 34.5%	-	-	-	-
			Provisional BP	163/207;	18/21; 85.7%	58/87; 66.7%	-	58/87; 66.7%	-	-	-	-
[126]	UK	2013–2016	EUCAST	276/342; 80.7%	7/24; 29.2%	144/217; 66.3%	7/24; 29.2%	103/164; 62.8%	0/4; 0%	38/40; 95%	7/20; 35%	3/13; 23%
			CLSI	240/342; 70.2%	7/24; 29.2%	123/217; 56.7%	7/24; 29.2%	76/164; 46.3%	0/4; 0%	37/40; 92.5%	7/20; 35%	0/13; 0%
			Provisional BP	304/342	10/24; 41.7%	180/217; 82.9%	10/24; 41.7%	131/164; 79.9%	0/4; 0%	40/40; 100%	10/20; 50%	9/13; 69.2%
[127]	Greece	2019–2020	EUCAST	78/97	46/100, 46%	78/97; 80.4%	46/100; 46%	-	-	-	-	-
			CLSI	61/97; 62.9%	46/100; 46%	61/97; 62.9%	46/100; 46%	-	-	-	-	-
			Provisional BP	89/97	89/100; 89%	89/97	89/100	-	-	-	-	-
[128]	Spain	2018	EUCAST	388/400	-	48/56; 85.7%	-	6/10; 60%	-	40/42; 95.2%	-	2/4; 50%
			CLSI	360/400; 90%	-	42/56; 75%	-	5/10; 50%	-	37/42; 88.1%	-	0/4; 0%
			Provisional BP	398/400	-	54/56; 96.4%	-	9/10; 90%		41/42; 97.6%		4/4; 100%
[129]	Spain	2020	EUCAST	229/247; 92.7%	115/170; 67.6%	38/45; 84.4%	25/53; 47.2%	2/4; 50%	-	36/39; 92.3%	25/45; 55.5%	0/2; 0%
			CLSI	207/247; 83.8%	115/170; 67.6%	34/45; 77.8%	25/53; 47.2%	0/4; 0%	-	34/39; 87.2%	25/45; 55.5%	0/2; 0%
			Provisional BP	245/247	147/170; 86.5%	43/45; 95.5%	35/53; 66%	4/4; 100%	-	38/39; 97.4%	35/45; 77.8%	1/2; 50%
[130]	India	2019–2021	EUCAST	209/570	-	14/250; 5.6%	-	14/250; 5.6%	-	-	-	-
			CLSI	172/570; 30.2%	-	12/250; 4.8%	-	12/250; 4.8%	-	-	-	-
			Provisional BP	338/570	-	70/250; 28%	-	70/250; 28%	-	-	-	-
Pooled data		2005–2023	EUCAST	2044/2693; 75.9%	475/750; 63.3%	560/1038; 53.9%	239/410; 58.3%	329/758; 43.4%	0/6; 0%	146/158; 92.4%	172/253; 68%	5/19; 26.3%
			CLSI	1798/2693; 66.8%	475/750; 63.3%	487/1035; 47%	239/410; 58.3%	276/758; 36.4%	0/6; 0%	138/158; 87.3%	172/253; 68%	0/19; 0%
			Provisional BP	2336/2693; 86.7%	615/750; 82%	748/1035; 72.3%	317/410; 77.3%	486/758; 64.1%	0/6; 0%	156/158; 98.7%	206/253; 81.4%	14/19; 73.7%

For susceptibility testing purpose, the concentration of taniborbactam was fixed at 4 mg/L

Susceptibility data were interpreted according to following breakpoints:

EUCAST cefepime susceptibility breakpoint (v_14.0, 2024): Enterobacterales, ≤ 4 mg/L; *Pseudomonas*, ≤ 8 mg/L

CLSI cefepime susceptibility breakpoints (CLSI M100 ED34:2024): Enterobacterales, ≤ 2 mg/L; *Pseudomonas*, ≤ 8 mg/L

Provisional cefepime/taniborbactam susceptibility breakpoint: ≤ 16 mg/L [124]

Abbreviation: BP, breakpoint

The phase 3 trial CERTAIN-1 (NCT03840148; registration date: 2019–02–06; https://clinicaltrials.gov/study/NCT03840148) compared efficacy and safety of cefepime/taniborbactam with meropenem for the treatment of adults with cUTI not caused by MβL-producing bacteria [137]. Cefepime/taniborbactam showed higher microbiological and clinical success than meropenem (treatment difference, 12.6%; 95% confidence interval, 3.1 to 22.2; p = 0.009) [137]. Another phase 3 clinical trial on efficacy and safety of cefepime/taniborbactam is ongoing (NCT06168734; registration date: 2023–12-04; https://clinicaltrials.gov/study/NCT06168734).

### β-lactam/xeruborbactam

Xeruborbactam (formerly QPX7728, Qpex Biopharma) is a bicyclic boronate-based β-lactamase inhibitor that shows ultrabroad-spectrum activity against all classes of β-lactamases (Fig. 2 and Table 1) [120, 138]. It was recently discovered in a project involving modification of boric acid pharmacophore to expand β-lactamase inhibition spectrum and achieve oral bioavailability [139]. Although its binding mode resembles that of taniborbactam, the introduction of a cyclopropyl group into the xeruborbactam structure enhances the hydrophobic interaction in the active site and the inhibitory activity. Xeruborbactam showed a potent inhibitory activity against class A extended-spectrum β-lactamases (CTX-M, SHV, TEM, VEB, PER) and carbapenemases (KPC, SME, NMC-A, BKC-1), plasmid-determined (CMY, FOX, MIR, DHA) and chromosomally encoded (P99, PDC, ADC) class C β-lactamases, class D enzymes, including OXA-48-like and OXA enzymes from *A. baumannii* (OXA-23/24/72/58), as well as various class B1 MβLs (NDM, VIM, CcrA, IMP, and GIM but not SPM or L1) [139, 140]. Despite xeruborbactam has similar relative inhibitory concentrations to taniborbactam against NDM and VIM enzymes, it showed being effective against taniborbactam resistant enzymes, such as NDM-9, NDM-30, VIM-83 and most of IMP enzymes [141].

Data on in vitro activity of xeruborbactam in combination with β-lactams are limited, and mainly involving meropenem/xeruborbactam combination [142–147]. Data on in vitro activity of meropenem/xeruborbactam against surveillance Gram-negative isolates, including MβL-producers, were reported in Table 5. Overall, potent in vitro activity was shown for carbapenem-resistant and/or carbapenemase-producing Enterobacterales (n = 1625) (> 94% of susceptibility), MβL-producing Enterobacterales (n = 534), and carbapenem-resistant and/or carbapenemase-producing *A. baumannii complex* isolates (n = 275) (> 95% of susceptibility). Lower susceptibility rates and higher MIC_50_/MIC_90_ values were observed in *P. aeruginosa*, especially among isolates resistant to carbapenems and/or ceftazidime/avibactam and/or ceftolozane/tazobactam (n = 290) (MIC_50_/MIC_90_ 8/64, 60.3% of susceptibility), and among MβL-producing isolates (n = 61) (MIC_50_/MIC_90_ 32/ > 64, 31.1% of susceptibility). Le Terrier et al*.* showed that xeruborbactam was less active than taniborbactam to reduce MIC values of β-lactams in MβL-producing *P. aeruginosa* recombinant strains, and this was caused by the activity of MexAB-OprM efflux pump [141].

**Table 5:** In vitro activity of meropenem/xeruborbactam against MDR Gram-negative clinical isolates collections including metallo-β-lactamase producers

References	Origin of isolates	Period of isolates collection	Fixed concentration of xeruborbactam	Bacterial species	MIC_50_/MIC_90_ (mg/L), susceptibility % (n° of isolates tested)				
				Enterobacterales					
					Carbapenem non-susceptible and/or carbapenemase-producers	MBL-producers	NDM-producers	VIM-producers	IMP-producers
[142]	Worldwide	2001–2017	4 mg/L		≤ 0.06/4, 96.5% (n = 598)	≤ 0.06/4, 95.5% (n = 224)	≤ 0.06/4, 94.7% (n = 151)	≤ 0.06/2, 98.1% (n = 53)	≤ 0.06/4, 95% (n = 20)
			8 mg/L		≤ 0.06/0.5, 99.3% (n = 598)	≤ 0.06/1; 98.2% (n = 224)	≤ 0.06/2, 98% (n = 151)	≤ 0.06/0.5, 100% (n = 53)	≤ 0.06/2, 95% (n = 20)
[143]	Worldwide	2018–2020	4 mg/L		0.06/0.5, 98.3% (n = 1027)	0.06/4, 95.8% (n = 310)	0.06/4, 95.8% (n = 287)	≤ 0.03/0.5, 100% (n = 20)	
			8 mg/L		≤ 0.03/0.25, 99.6% (n = 1027)	≤ 0.03/1, 98.7% (n = 310)	≤ 0.03/1, 99% (n = 287)	≤ 0.03/0.06, 100% (n = 20)	
				*A. baumannii-calcoaceticus* complex					
					Carbapenem-resistant	NDM-producers			
[144]	Worldwide	1998–2018	4 mg/L		2/8 mg/L, 94.5% (n = 275)	60% (n = 5)			
			8 mg/L		1/4 mg/L, 98.5% (n = 275)	100% (n = 5)			
				*P. aeruginosa*					
					Overall isolates	DTT *isolates*	MβL-producers		
[145]	Worldwide	2016–2018	8 mg/L		0.25/8, 91.6% (n = 500)	8/64, 60.3% (n = 290)	32/ > 64, 31.1% (n = 61)		

For susceptibility testing purpose, the concentration of xeruborbactam was fixed at 4 mg/L or 8 mg/L

No available clinical breakpoints for meropenem/xeruborbactam. Susceptibility data were interpreted according to EUCAST/CLSI (2024) meropenem susceptibility breakpoints: ≤ 8 mg/L

MIC_50_/MIC_90_ values were not reported for isolates number ≤ 10

Abbreviations: DTT, difficult to treat

Data on in vivo efficacy of β-lactam/xeruborbactam combinations are lacking. Currently, phase 1 clinical studies on xeruborbactam in combination with ceftibuten are ongoing to evaluate the safety and pharmacokinetics of orally administered treatments (NCT06079775; registration date: 2023–10-06; https://clinicaltrials.gov/study/NCT06079775; and NCT06157242; registration date: 2023–11–27; https://clinicaltrials.gov/study/NCT06157242)**.** In addition, a recently registered phase 1 clinical study (NCT06547554; registration date: 2024–08-02; https://clinicaltrials.gov/study/NCT06547554) aims at evaluating the combination cefiderocol/xeruborbactam in healthy adults.

### Cefepime/zidebactam

Zidebactam (formerly WCK 5107; Wockhardt, Aurangabad, India) is a diazabicyclooctane β-lactamase inhibitor, with PBP-2 binding activity (Fig. 2) [148]. Combination of zidebactam with cefepime (formerly WCK 5222) represents the first β-lactam/β-lactamase inhibitor combination that elicits rapid bactericidal activity at the sub-MIC level through the simultaneous inactivation of PBP-2 (zidebactam) and PBP-3 (cefepime) (Table 1). The enhancement of cefepime activity by high-affinity binding of PBP-2 by zidebactam occurs independently of β-lactamase expression. Therefore, this combination is different from the previous ones that merely preserve the activity of β-lactam antibiotic partners. Moreover, zidebactam is reported to inhibit several class A and class C β-lactamases and some class D enzymes [148]. Thus, the cefepime plus zidebactam offers a potential treatment for the infections caused by cefepime-resistant Gram-negative bacilli isolates, carbapenem-resistant isolates (KPC or MBL-producing), and for many other MDR isolates [63, 128, 130, 149–156]. Data on in vitro activity of cefepime/zidebactam (tested at ratio 1:1) were reported in Table 6. Cefepime/zidebactam showed high activity towards carbapenem-resistant and/or carbapenemase-producing Enterobacterales (90.6–98%) and *P. aeruginosa* (89.4–99.1%). Moreover, high in vitro activity was shown towards MβL-producers [83.4–95.3% and 83.9–96.4%, in Enterobacterales (n = 1326) and *P. aeruginosa* (n = 338), respectively]. Conversely, significant discrepancy in susceptibility rates (95.7% *vs.* 24.9%) was observed in carbapenem-resistant and/or carbapenemase-producing *A. baumannii* using the provisional PK/PD susceptibility breakpoint (≤ 64 mg/L) and the CLSI susceptibility breakpoint of cefepime (≤ 8 mg/L), respectively.

**Table 6:** In vitro activity of cefepime/zidebactam against MDR Gram-negative clinical isolates collections including metallo-β-lactamase producers

References	Country	Period of isolates collection	Breakpoint	Carbapenem non-susceptible and/or carbapenemase-producers			MβL producers			NDM producers
				Enterobacterales	*P. aeruginosa*	ACB	Enterobacterales	*P. aeruginosa*	ACB	Enterobacterales
[63]	Swiss	2022–23	EUCAST/CLSI	-	21/39; 53.8%	-	-	21/39; 53.8%	-	-
			Provisional BP	-	28/39; 71.8%	-	-	28/39; 71.8%	-	-
[128]	Spain	2018	EUCAST	398/400; 99.5%	-	-	52/56; 92.8%	-	-	10/10; 100%
			CLSI	384/400; 99.5%	-	-	46/56; 82.1%	-	-	10/10; 100%
			Provisional BP	400/400; 100%	-	-	54/56; 96.4%	-	-	10/10; 100%
[130]	India	2019–2021	EUCAST	553/569; 97.2%	-	-	402/418; 96.2%	-	-	-
			CLSI	529/569; 93%	-		379/418; 90.7%	-	-	-
			Provisional BP	569/569; 100%	-		418/418; 100%	-	-	-
[150]	Worldwide	-	EUCAST	984/1018; 96.7%	157/262; 59.9%	-	-	-	-	-
			CLSI	896/1018; 88%	157/262; 59.9%	-	-	-	-	-
			Provisional BP/PKPD	1003/1018; 98.5%	261/262; 99.6%	-	203/214; 94.8%	94/94; 100%	-	-
[151]	Taiwan	2012–2018	EUCAST	-	74/81; 91.3%	-	-	3/4; 75%	-	-
			CLSI	-	74/81; 91.3%	11/135; 8.1%	-	3/4; 75%	-	-
			Provisional BP	179/180; 99.4%	-	-	92/92; 100%	-	-	-
[152]	UK	2015–2016	EUCAST	568/619; 91.8%	91/96; 94.8%	-	183/234; 78.2%	76/81; 93.8%	-	-
			CLSI	536/619; 86.6%	91/96; 94.8%	98/202; 48.5%	155/234; 66.2%	76/81; 93.8%	0/19; 0%	-
			Provisional BP/PKPD	586/619; 86.6%	96/96; 100%	188/202; 93.1%	201/234; 85.6%	81/81; 100%	6/19; 31.6%	-
[153]	Greek	2014–2018	EUCAST	406/422; 96.2%	154/172; 89.5%	-	176/186; 94.6%	93/106; 87.7%	-	-
			CLSI	391/422; 92.6%	154/172; 89.5%	20/181; 11%	166/186; 89.2%	93/106; 87.7%	-	-
			Provisional BP	415/422; 98.3%	171/172; 99.4%	174/181: 96.1%	182/186; 97.8%	105/106; 99%	-	-
[154]	China	2019	Provisional BP	364/379; 96%	224/228; 98.2%	455/471; 96.6%	114/126; 90.5%	-	-	105/117; 89.7%
[155]	Worldwide	2018–2019	EUCAST	656/681; 96.3%	1108/1147; 96.6%	-	-	-	-	-
			CLSI	626/681; 91.9%	1108/1147; 96.6%	-	-	-	-	-
			Provisional BP	666/681; 97.8%	1146/1147; 99.9%	-	-	-	-	-
[156]	USA	-	EUCAST/CLSI	-	98/108; 90.7%	-	-	15/18; 83.3%	-	-
			Provisional BP	-	108/108; 100%	-	-	18/18; 100%	-	-
Pooled data		2012–2023	EUCAST	3565/3709; 96.1%	1703/1905; 89.4%	-	813/894; 90.9%	208/248; 83.9%	-	10/10; 100%
			CLSI	3362/3709; 90.6%	1703/1905; 89.4%	129/518; 24.9%	746/894; 83.4%	208/248; 83.9%	0/19; 0%	10/10; 100%
			Provisional BP	4182/4268; 98%	2034/2052; 99.1%	817/854; 95.7%	1264/1326; 95.3%	326/338; 96.4%	6/19; 31.6%	115/127; 90.5%

Cefepime and zidebactam were tested at a ratio of 1:1

Susceptibility data were interpreted using EUCAST (2024), CLSI (2024) and provisional breakpoints [150] as follows:

EUCAST cefepime susceptibility breakpoint (v_14.0, 2024): Enterobacterales, ≤ 4 mg/L; *Pseudomonas*, ≤ 8 mg/L

CLSI cefepime susceptibility breakpoints (CLSI M100 ED34:2024): Enterobacterales, ≤ 2 mg/L; *Pseudomonas*, ≤ 8 mg/L; *Acinetobacter*, ≤ 8 mg/L;

Provisional cefepime/zidebactam susceptibility breakpoints: Enterobacterales, ≤ 8 mg/L; *Pseudomonas* (PK/PD breakpoint) ≤ 32 mg/L; *Acinetobacter* (PK/PD breakpoint) ≤ 64 mg/L

Abbreviation: ACB, *Acinetobacter baumannii*-*calcoaceticus* complex; BP, breakpoint

Excellent in vitro activity of cefepime/zidebactam was shown against ceftazidime/avibactam and ceftolozane/tazobactam resistant *P. aeruginosa* [157]. Moreover, high activity with both MIC_50_ and MIC_90_ at 0.25 mg/L was observed in aztreonam/avibactam and cefepime/taniborbactam resistant *E.coli* strains harboring NDM-variants (NDM-1, NDM-4, NDM-5), CMY-42 and mutated PBP-3 [158].

Inactivation of serine-β-lactamases combined with the direct antibacterial effect of zidebactam results in modest impact of β-lactamases, including double carbapenemase production [141, 147]. Moreover, no impact on resistance was observed in Omp-deficient *E. coli* and *K. pneumoniae,* suggesting synergistic activity of cefepime and zidebactam overcomes mechanisms affecting cell permeability [147, 159–162]. On the other hand, resistance to cefepime/zidebactam required multiple mutations in genes encoding MexAB-OprM and its regulators, as well as PBP-2 and PBP-3 [159–163]. PBP-2 is a transpeptidase that is involved in peptidoglycan cross-linking and cell wall elongation. Inhibition of PBP-2 by zidebactam leads to round cell formation [164, 165]. Resistance to zidebactam was shown to be due to missense mutations in the transpeptidase domain of *pbpA* gene (from D351 to V598) and the I450 position involved in direct interaction with zidebactam [159, 160]. Insertion of IS*Pa1635* in IS*CR1* upstream of *bla*_PER-1_ resulted in elevated transcription of *bla*_PER-1_ and increased resistance to ceftazidime/avibactam, ceftolozane/tazobactam and cefepime/zidebactam in a *P. aeruginosa* clinical strain [166].

Translational in vivo studies in neutropenic mice lung or thigh models showed efficacy of cefepime/zidebactam against MβL-expressing *P. aeruginosa* and carbapenemase-producing *K. pneumoniae* at mimicking human exposures [167–169]. Cefepime/zidebactam is currently under evaluation in a global phase 3 trial in adult patients with cUTI or acute pyelonephritis (NCT04979806; registration date: 2021–07-05; https://www.clinicaltrials.gov/study/NCT04979806). Successful compassionate use in treating NDM-producing *P. aeruginosa* infections was already reported [170–172].

### β-lactam/nacubactam

Nacubactam (formerly RG6080/OP0595; Roche, Fedora, Meiji) is a new diazabicyclooctane β-lactamase inhibitor that inhibits various types of β-lactamases, including Ambler class A, class C, and class D (OXA-48) β-lactamases (Fig. 2 and Table 1). Similarly to zidebactam, nacubactam has significant affinity for PBP-2 of many Gram-negative species, allowing it to exert both a direct antibacterial effect and enhancing partner β-lactams that bind to PBP-3 [173, 174].

IC_50_ values of nacubactam for representative class A and C β-lactamases were similar to those of avibactam or slightly higher. Conversely, class D β-lactamases, and particularly OXA-23, appeared more resistant to inhibition [173]. These characteristics allowed to consider nacubactam in combination with various β-lactam agents (meropenem, cefepime, aztreonam) as a potential drug against MDR Gram-negative bacteria, including MβL-producers. Data on in vitro activity of β-lactam/nacubactam combinations are very limited [173–175].

Meropenem/nacubactam and cefepime/nacubactam showed high activity against MβL producing Enterobacterales (NDM, n = 158; VIM, n = 52; IMP, n = 99), regardless both MβL type and aztreonam-resistance status [174]. In detail, meropenem/nacubactam at 8 + 4 mg/L and cefepime/nacubactam at 8 + 4 mg/L were active against 87.1% and 93.3% of isolates tested [174]. Terrier et al. also evaluated in vitro activity of aztreonam in combination with novel β-lactamase inhibitors (at fixed concentration 4 mg/L) and cefiderocol against Enterobacterales (n = 64) and *P. aeruginosa* (n = 39) clinical isolates producing representative MβLs [NDM (n = 64), VIM (n = 32), IMP (n = 8) and SPM (n = 2)]. Among Enterobacterales isolates, aztreonam/zidebactam showed the highest activity (98.4%), followed by aztreonam/nacubactam (84.4%), aztreonam/taniborbactam (75%), aztreonam/avibactam (70.3%) and cefiderocol (39.1%). Lower activity was observed against MβL-producing *P. aeruginosa* isolates, with susceptibility rates of 66.7% for aztreonam/nacubactam and aztreonam/taniborbactam, and 69.2% with aztreonam/avibactam, aztreonam/zidebactam and cefiderocol [175]. These findings could be due to low intrinsic activity of nacubactam against *P. aeruginosa*, owing to the higher intrinsic resistance of this pathogen (MICs of 32 mg/L when tested alone) [173]. Moreover, common resistance mechanisms in *P. aeruginosa* such as *mexAB-oprM* overexpression and OprD deficiency, or increased expression of *bla*_PDC_ have been associated to resistance to meropenem-based combinations, including meropenem/nacubactam [161].

Moreover, since nacubactam as well as zidebactam targets PBP-2, mutations in *pbpA* gene are expected to be involved in resistance in both Enterobacterales and *Pseudomonas* species [159, 160].

Nacubactam combined with β-lactams (meropenem, cefepime, aztreonam) showed high in vivo antimicrobial activity in murine model against carbapenem-resistant and carbapenemase (including MβL)-producing *E. coli* and *K. pneumoniae* [176–178]. Safety profile of meropenem/nacubactam and favorable pharmacokinetic parameters were reported in healthy adults [179]. Two phase 3 trials evaluating safety and efficacy of nacubactam combined with cefepime and aztreonam for the treatment of cUTI or acute uncomplicated pyelonephritis caused by carbapenem-resistant Enterobacterales have been registred (NCT05887908; registration date: 2023–04–25; https://clinicaltrials.gov/study/NCT05887908; and NCT05905055 registration date: 2023–03-02; https://clinicaltrials.gov/study/NCT05905055).

### Sulbactam/durlobactam

Sulbactam/durlobactam (XACDURO®, Entasis Therapeutics), was approved in May 2023 by the U.S. Food and Drug Administration for the treatment of adult patients with HAP/VAP caused by susceptible isolates of *A. baumannii* complex (Table 1) [180, 181].

Sulbactam (a penicillin derivative) is a β-lactam antibacterial and Ambler class A serine β-lactamase inhibitor that also has bactericidal activity due to its inhibition of PBP-1 and PBP-3 [182]. Durlobactam (formerly ETX2514, Entasis Therapeutics) is a next generation diazabicyclooctane β-lactamase inhibitor with potent activity against class A, C, and D serine β-lactamases and intrinsic antibacterial activity on PBP-2 (Fig. 2 and Table 1) [183]. However, PBP-2 inhibition by durlobactam resulted in intrinsic antibacterial activity against *E. coli* and several other Enterobacterales species, but it has little to no effect on the growth of *A. baumannii* or *P. aeruginosa* when administered alone [184]. The key feature as compared to zidebactam and nacubactam is its activity against class D carbapenemases of the OXA family, which are prevalent in *A. baumannii* [184]. Hence, combination of durlobactam to sulbactam was reported to lower MIC_90_ by 32-fold (from 64 mg/L to 2 mg/L) compared to sulbactam alone in *A. baumannii* [185], resulting in high susceptibility rates (> 97%) in global collections of MDR *A. baumannii* clinical isolates [185, 186]. Furthermore, clinical efficacy was shown in the phase 3 ATTACK clinical trial, in which sulbactam/durlobactam was observed to be non-inferior to colistin for the treatment of patients with severe infections caused by *A. baumannii* complex [181].

Resistance to sulbactam/durlobactam in *A. baumannii* was associated with both expression of MβLs towards which durlobactam has no inhibitory activity and alteration in PBP-3 and/or PBP-2 [185, 186]. Potent intrinsic activity of durlobactam on PBP-2 of Enterobacterales and its stability to the hydrolytic action of β-lactamases represent an interesting therapeutic potential towards MDR strains including those producing MBLs [187]. A recent report showed high activity of sulbactam/durlobactam against NDM-producing *E. coli*, including several MβL variants (e.g. NDM-5, NDM-1, NDM-7) and strains harboring PBP-3 modifications leading to resistance to aztreonam/avibactam and/or cefiderocol [188]. These findings could legitimize future investigations on sulbactam/durlobactam role in the clinical management of infections sustained by MβL-producing Enterobacterales.

## Conclusions

The recent development of new antimicrobials expanded the armamentarium to counter the challenge of MβL-producers. Cefiderocol and aztreonam/avibactam are already available. In addition, two new classes of β-lactam/β-lactamase combinations are under clinical evaluation: (*i*) combination of β-lactam with novel MβL inhibitors (taniborbactam and xeruborbactam), (*ii*) combination of β-lactam with new diazabicyclooctane β-lactamase inhibitors, active on most of serine-β-lactamase but also showing strong intrinsic activity on PBP-2.

In vitro activity of aztreonam/avibactam against MβL-producing Enterobacterales is higher than that of cefiderocol, providing supporting evidence on its key role in the treatment of infections sustained by these strains. On the other hand, aztreonam/avibactam does not show satisfactory activity against MβL-producing *P. aeruginosa* and MDR *A. baumannii* given their ability to display multiple resistance mechanisms. Therefore, in these contexts, cefiderocol may represent a more appropriate therapeutic option, given the excellent activity observed with the exception of some NDM-producing clones. Both cefiderocol and aztreonam/avibactam showed high in vitro activity against *S. maltophilia*, an emerging nosocomial MDR pathogen expressing the L1 chromosomal MβL.

In the group of β-lactam/new MβL inhibitor combinations, cefepime/taniborbactam showed potent activity against MβL-producing Enterobacterales, especially VIM-producing strains. The main limitation is the poor activity of taniborbactam towards IMP-carbapenemases, VIM-83 and some NDM-variants (NDM-9, NDM-30). This limitation is overcome by the xeruborbactam, which has a wide inhibition spectrum, including OXA-23-like carbapenemases commonly expressed by *A. baumannii* isolates. Despite these features, taniborbactam- and xeruborbactam-based combinations, offer a more limited therapeutic opportunity against *P. aeruginosa* given thecommon mechanisms of upregulation of efflux pumps, permeability loss and AmpC beta-lactamase overespression found in this species. Activity of these new combinations, as well as those of cefiderocol and aztreonam/avibactam, are affected by mutations in PBP-3, which is the target of the β-lactam molecule but this could be bypassed by the combinations of β-lactam with new diazabicyclooctane β-lactamase inhibitors nacubactam and zidebactam. This effective strategy has feedback on in vitro activity, especially for cefepime/zidebactam, against MDR Enterobacterales, *P. aeruginosa*, and *A. baumannii* complex isolates, including MβL-producing ones.

Future studies should evaluate the possibility of combining cefiderocol with the new β-lactamase inhibitors (xeruborbactam and zidebactam) investigating the feasibility of new synergistic strategies. Given the presence of resistance mechanisms and the possibility of selection of mutant strains during therapy, the appropriate use of these new drugs should require the availability of commercial assays for in vitro susceptibility testing, which would allow the implementation of surveillance programmes appropriate to the complexity of the phenomenon.

## Data Availability

No datasets were generated or analysed during the current study.

## References

[1] Murray CJL, Ikuta KS, Sharara F, Swetschinski L, Robles Aguilar G, Gray A et al (2022) Global burden of bacterial antimicrobial resistance in 2019: a systematic analysis. Lancet 399(10325):629–655. 10.1016/S0140-6736(21)02724-0 35065702 10.1016/S0140-6736(21)02724-0 PMC8841637

[2] O'Neill J (2016) Tackling drug-resistant infections globally: final report and recommendations. Review on Antimicrobial Resistance. 10.5555/20173071720

[3] Founou RC, Blocker AJ, Noubom M, Tsayem C, Choukem SP, Dongen MV, Founou LL (2021) The COVID-19 pandemic: a threat to antimicrobial resistance containment. Future Sci OA 7(8):FSO736. 10.2144/fsoa-2021-0012 34290883 10.2144/fsoa-2021-0012 PMC8204817

[4] Casale R, Bianco G, Bastos P, Comini S, Corcione S, Boattini M, Cavallo R, Rosa FG, Costa C (2023) Prevalence and impact on mortality of colonization and super-infection by carbapenem-resistant gram-negative organisms in COVID-19 hospitalized patients. Viruses 15:1934. 10.3390/v15091934 37766340 10.3390/v15091934 PMC10534345

[5] Ajulo S, Awosile B (2024) Global antimicrobial resistance and use surveillance system (GLASS 2022): investigating the relationship between antimicrobial resistance and antimicrobial consumption data across the participating countries. PLoS ONE 19:e0297921. 10.1371/journal.pone.0297921 38315668 10.1371/journal.pone.0297921 PMC10843100

[6] GBD 2021 Antimicrobial Resistance Collaborators (2024) Global burden of bacterial antimicrobial resistance 1990–2021: a systematic analysis with forecasts to 2050. Lancet 404(10459): 1199-1226. 10.1016/S0140-6736(24)01867-1 10.1016/S0140-6736(24)01867-1 PMC11718157 39299261

[7] Bonomo RA, Burd EM, Conly J, Limbago BM, Poirel L, Segre JA, Westblade LF (2018) Carbapenemase-producing organisms: a global scourge. Clin Infect Dis 66:1290–1297. 10.1093/cid/cix893 29165604 10.1093/cid/cix893 PMC5884739

[8] Papadimitriou-Olivgeris M, Bartzavali C, Lambropoulou A, Solomou A, Tsiata E, Anastassiou ED, Fligou F, Marangos M, Spiliopoulou I, Christofidou M (2019) Reversal of carbapenemase-producing Klebsiella pneumoniae epidemiology from blaKPC- to blaVIM-harbouring isolates in a Greek ICU after introduction of ceftazidime/avibactam. J Antimicrob Chemother 74:2051–2054. 10.1093/jac/dkz125 31002313 10.1093/jac/dkz125

[9] Qu J, Feng C, Li H, Lv X (2021) Antibiotic strategies and clinical outcomes for patients with carbapenem-resistant Gram-negative bacterial bloodstream infection. Int J Antimicrob Agents 57:106284. 10.1016/j.ijantimicag.2021.106284 33484833 10.1016/j.ijantimicag.2021.106284

[10] Nordmann P, Poirel L (2002) Emerging carbapenemases in Gram-negative aerobes. Clin Microbiol Infect 8:321–331. 10.1046/j.1469-0691.2002.00401.x 12084099 10.1046/j.1469-0691.2002.00401.x

[11] Salahuddin P, Kumar A, Khan AU (2018) Structure, function of serine and metallo-β-lactamases and their inhibitors. Curr Protein Pept Sci 19:130–144. 10.2174/0929866524666170724160623 28745223 10.2174/0929866524666170724160623

[12] Oelschlaeger P, Kaadan H, Dhungana R (2023) Strategies to name metallo-β-lactamases and number their amino acid residues. Antibiotics (Basel) 12:1746. 10.3390/antibiotics12121746 38136780 10.3390/antibiotics12121746 PMC10740994

[13] Naas T, Oueslati S, Bonnin RA, Dabos ML, Zavala A, Dortet L, Retailleau P, Iorga BI (2017) Beta-lactamase database (BLDB) - structure and function. J Enzyme Inhib Med Chem 32:917–919. 10.1080/14756366.2017.1344235 28719998 10.1080/14756366.2017.1344235 PMC6445328

[14] Casale R, Boattini M, Comini S, Bastos P, Corcione S, De Rosa FG, Bianco G, Costa C (2024) Clinical and microbiological features of positive blood culture episodes caused by non-fermenting gram-negative bacilli other than Pseudomonas and Acinetobacter species (2020–2023). Infection. 10.1007/s15010-024-02342-6 38990473 10.1007/s15010-024-02342-6 PMC11825528

[15] Mojica MF, Rossi MA, Vila AJ, Bonomo RA (2022) The urgent need for metallo-β-lactamase inhibitors: an unattended global threat. Lancet Infect Dis 22:e28–e34. 10.1016/S1473-3099(20)30868-9 34246322 10.1016/S1473-3099(20)30868-9 PMC8266270

[16] Cheng Z, Thomas PW, Ju L, Bergstrom A, Mason K, Clayton D, Miller C, Bethel CR, VanPelt J, Tierney DL, Page RC, Bonomo RA, Fast W, Crowder MW (2018) Evolution of New Delhi metallo-β-lactamase (NDM) in the clinic: Effects of NDM mutations on stability, zinc affinity, and mono-zinc activity. J Biol Chem 293:12606–12618. 10.1074/jbc.RA118.003835 29909397 10.1074/jbc.RA118.003835 PMC6093243

[17] Bahr G, Vitor-Horen L, Bethel CR, Bonomo RA, González LJ, Vila AJ (2017) Clinical evolution of New Delhi metallo-β-lactamase (NDM) optimizes resistance under Zn(II) deprivation. Antimicrob Agents Chemother 62:e01849-e1917. 10.1128/AAC.01849-17 29038264 10.1128/AAC.01849-17 PMC5740384

[18] Watanabe M, Iyobe S, Inoue M, Mitsuhashi S (1991) Transferable imipenem resistance in Pseudomonas aeruginosa. Antimicrob Agents Chemother 35:147–151. 10.1128/AAC.35.1.147 1901695 10.1128/aac.35.1.147 PMC244956

[19] Hupková M, Blahová J, Babalova M, Krcméry V, Schäfer V (1993) Transferable resistance to imipenem in hospital isolates of Pseudomonas aeruginosa. J Chemother 5:14–16. 10.1080/1120009x.1993.11739202 8459259 10.1080/1120009x.1993.11739202

[20] Arakawa Y, Murakami M, Suzuki K, Ito H, Wacharotayankun R, Ohsuka S, Kato N, Ohta M (1995) A novel integron-like element carrying the metallo-beta-lactamase gene blaIMP. Antimicrob Agents Chemother 39:1612–1615. 10.1128/AAC.39.7.1612 7492116 10.1128/aac.39.7.1612 PMC162793

[21] Ito H, Arakawa Y, Ohsuka S, Wacharotayankun R, Kato N, Ohta M (1995) Plasmid-mediated dissemination of the metallo-beta-lactamase gene blaIMP among clinically isolated strains of Serratia marcescens. Antimicrob Agents Chemother 39:824–829. 10.1128/AAC.39.4.824 7785978 10.1128/aac.39.4.824 PMC162636

[22] Koh TH, Babini GS, Woodford N, Sng LH, Hall LM, Livermore DM (1999) Carbapenem-hydrolysing IMP-1 beta-lactamase in Klebsiella pneumoniae from Singapore. Lancet 353:2162. 10.1016/s0140-6736(05)75604-x 10382730 10.1016/s0140-6736(05)75604-x

[23] Senda K, Arakawa Y, Nakashima K, Ito H, Ichiyama S, Shimokata K, Kato N, Ohta M (1996) Multifocal outbreaks of metallo-beta-lactamase-producing Pseudomonas aeruginosa resistant to broad-spectrum beta-lactams, including carbapenems. Antimicrob Agents Chemother 40:349–353. 10.1128/AAC.40.2.349 8834878 10.1128/aac.40.2.349 PMC163114

[24] Cornaglia G, Riccio ML, Mazzariol A, Lauretti L, Fontana R, Rossolini GM (1999) Appearance of IMP-1 metallo-beta-lactamase in Europe. Lancet 353:899–900. 10.1016/s0140-6736(98)05954-6 10093989 10.1016/s0140-6736(98)05954-6

[25] Riccio ML, Franceschini N, Boschi L, Caravelli B, Cornaglia G, Fontana R, Amicosante G, Rossolini GM (2000) Characterization of the metallo-beta-lactamase determinant of Acinetobacter baumannii AC-54/97 reveals the existence of bla(IMP) allelic variants carried by gene cassettes of different phylogeny. Antimicrob Agents Chemother 44:1229–1235. 10.1128/AAC.44.5.1229-1235.2000 10770756 10.1128/aac.44.5.1229-1235.2000 PMC89849

[26] Da Silva GJ, Correia M, Vital C, Ribeiro G, Sousa JC, Leitão R, Peixe L, Duarte A (2002) Molecular characterization of bla(IMP-5), a new integron-borne metallo-beta-lactamase gene from an Acinetobacter baumannii nosocomial isolate in Portugal. FEMS Microbiol Lett 215:33–39. 10.1111/j.1574-6968.2002.tb11366.x 12393197 10.1111/j.1574-6968.2002.tb11366.x

[27] Iyobe S, Kusadokoro H, Ozaki J, Matsumura N, Minami S, Haruta S, Sawai T, O’Hara K (2000) Amino acid substitutions in a variant of IMP-1 metallo-beta-lactamase. Antimicrob Agents Chemother 44:2023–2027. 10.1128/AAC.44.8.2023-2027.2000 10898670 10.1128/aac.44.8.2023-2027.2000 PMC90008

[28] Chu YW, Afzal-Shah M, Houang ET, Palepou MI, Lyon DJ, Woodford N, Livermore DM (2001) IMP-4, a novel metallo-beta-lactamase from nosocomial Acinetobacter spp. collected in Hong Kong between 1994 and 1998. Antimicrob Agents Chemother 45:710–4. 10.1128/AAC.45.3.710-714.2001 11181348 10.1128/AAC.45.3.710-714.2001 PMC90361

[29] Partridge SR, Ginn AN, Paulsen IT, Iredell JR (2012) pEl1573 Carrying blaIMP-4, from Sydney, Australia, is closely related to other IncL/M plasmids. Antimicrob Agents Chemother 56:6029–6032. 10.1128/AAC.01189-12 22926566 10.1128/AAC.01189-12 PMC3486572

[30] Sidjabat HE, Heney C, George NM, Nimmo GR, Paterson DL (2014) Interspecies transfer of blaIMP-4 in a patient with prolonged colonization by IMP-4-producing enterobacteriaceae. J Clin Microbiol 52:3816–3818. 10.1128/JCM.01491-14 25056334 10.1128/JCM.01491-14 PMC4187761

[31] Pongchaikul P, Mongkolsuk P (2022) Comprehensive analysis of imipenemase (IMP)-type metallo-β-lactamase: a global distribution threatening asia. Antibiotics (Basel) 11:236. 10.3390/antibiotics11020236 35203838 10.3390/antibiotics11020236 PMC8868347

[32] Hansen GT (2021) Continuous evolution: perspective on the epidemiology of carbapenemase resistance among enterobacterales and other gram-negative bacteria. Infect Dis Ther 10:75–92. 10.1007/s40121-020-00395-2 33492641 10.1007/s40121-020-00395-2 PMC7954928

[33] Bush K, Bradford PA (2020) Epidemiology of β-lactamase-producing pathogens. Clin Microbiol Rev 33(2):e00047-e119. 10.1128/CMR.00047-19 32102899 10.1128/CMR.00047-19 PMC7048014

[34] Ghaith DM, Zafer MM, Ismail DK, Al-Agamy MH, Bohol MFF, Al-Qahtani A, Al-Ahdal MN, Elnagdy SM, Mostafa IY (2018) First reported nosocomial outbreak of *Serratia marcescens* harboring *bla*_IMP-4_ and *bla*_VIM-2_ in a neonatal intensive care unit in Cairo, Egypt. Infect Drug Resist 11:2211–2217. 10.2147/IDR.S174869 30519059 10.2147/IDR.S174869 PMC6233950

[35] Yaghi J, Fattouh N, Akkawi C, El Chamy L, Maroun RG, Khalil G (2020) Unusually high prevalence of cosecretion of ambler class A and B carbapenemases and nonenzymatic mechanisms in multidrug-resistant clinical isolates of *Pseudomonas aeruginosa* in Lebanon. Microb Drug Resist 26:150–159. 10.1089/mdr.2019.0040 31424353 10.1089/mdr.2019.0040

[36] Bush K, Bradford PA (2020) Epidemiology of β-lactamase-producing pathogens. Clin Microbiol Rev 33:e00047-e119. 10.1128/CMR.00047-19 32102899 10.1128/CMR.00047-19 PMC7048014

[37] Lauretti L, Riccio ML, Mazzariol A, Cornaglia G, Amicosante G, Fontana R, Rossolini GM (1999) Cloning and characterization of blaVIM, a new integron-borne metallo-beta-lactamase gene from a Pseudomonas aeruginosa clinical isolate. Antimicrob Agents Chemother 43:1584–1590. 10.1128/AAC.43.7.1584 10390207 10.1128/aac.43.7.1584 PMC89328

[38] Poirel L, Naas T, Nicolas D, Collet L, Bellais S, Cavallo JD, Nordmann P (2000) Characterization of VIM-2, a carbapenem-hydrolyzing metallo-beta-lactamase and its plasmid- and integron-borne gene from a Pseudomonas aeruginosa clinical isolate in France. Antimicrob Agents Chemother 44:891–897. 10.1128/AAC.44.4.891-897.2000 10722487 10.1128/aac.44.4.891-897.2000 PMC89788

[39] Pournaras S, Maniati M, Petinaki E, Tzouvelekis LS, Tsakris A, Legakis NJ, Maniatis AN (2003) Hospital outbreak of multiple clones of Pseudomonas aeruginosa carrying the unrelated metallo-beta-lactamase gene variants blaVIM-2 and blaVIM-4. J Antimicrob Chemother 51:1409–1414. 10.1093/jac/dkg239 12716773 10.1093/jac/dkg239

[40] Cagnacci S, Gualco L, Roveta S, Mannelli S, Borgianni L, Docquier JD, Dodi F, Centanaro M, Debbia E, Marchese A, Rossolini GM (2008) Bloodstream infections caused by multidrug-resistant Klebsiella pneumoniae producing the carbapenem-hydrolysing VIM-1 metallo-beta-lactamase: first Italian outbreak. J Antimicrob Chemother 61:296–300. 10.1093/jac/dkm471 18065411 10.1093/jac/dkm471

[41] Kazmierczak KM, Rabine S, Hackel M, McLaughlin RE, Biedenbach DJ, Bouchillon SK, Sahm DF, Bradford PA (2015) Multiyear, multinational survey of the incidence and global distribution of metallo-β-lactamase-producing enterobacteriaceae and pseudomonas aeruginosa. Antimicrob Agents Chemother 60:1067–1078. 10.1128/AAC.02379-15 26643349 10.1128/AAC.02379-15 PMC4750703

[42] Ramirez MS, Bonomo RA, Tolmasky ME (2020) Carbapenemases: transforming *Acinetobacter baumannii* into a yet more dangerous menace. Biomolecules 10:720. 10.3390/biom10050720 32384624 10.3390/biom10050720 PMC7277208

[43] Yong D, Toleman MA, Giske CG, Cho HS, Sundman K, Lee K, Walsh TR (2009) Characterization of a new metallo-beta-lactamase gene, bla(NDM-1), and a novel erythromycin esterase gene carried on a unique genetic structure in Klebsiella pneumoniae sequence type 14 from India. Antimicrob Agents Chemother 53:5046–5054. 10.1128/AAC.00774-09 19770275 10.1128/AAC.00774-09 PMC2786356

[44] Wu W, Feng Y, Tang G, Qiao F, McNally A, Zong Z (2019) NDM metallo-β-lactamases and their bacterial producers in health care settings. Clin Microbiol Rev 32:e00115-e118. 10.1128/CMR.00115-18 30700432 10.1128/CMR.00115-18 PMC6431124

[45] Karthikeyan K, Thirunarayan MA, Krishnan P (2010) Coexistence of blaOXA-23 with blaNDM-1 and armA in clinical isolates of Acinetobacter baumannii from India. J Antimicrob Chemother 65:2253–2254. 10.1093/jac/dkq273 20650909 10.1093/jac/dkq273

[46] Dadashi M, Yaslianifard S, Hajikhani B, Kabir K, Owlia P, Goudarzi M, Hakemivala M, Darban-Sarokhalil D (2019) Frequency distribution, genotypes and prevalent sequence types of New Delhi metallo-β-lactamase-producing Escherichia coli among clinical isolates around the world: a review. J Glob Antimicrob Resist 19:284–293. 10.1016/j.jgar.2019.06.008 31212107 10.1016/j.jgar.2019.06.008

[47] Aslan AT, Paterson DL (2024) Epidemiology and clinical significance of carbapenemases in Australia: a narrative review. Intern Med J 54:535–544. 10.1111/imj.16374 38584572 10.1111/imj.16374

[48] Wu Y, Chen J, Zhang G, Li J, Wang T, Kang W, Zhang J, Sun H, Liu Y, Xu Y (2024) In-vitro activities of essential antimicrobial agents including aztreonam/avibactam, eravacycline, colistin and other comparators against carbapenem-resistant bacteria with different carbapenemase genes: a multi-centre study in China, 2021. Int J Antimicrob Agents 64:107341. 10.1016/j.ijantimicag.2024.107341 39304121 10.1016/j.ijantimicag.2024.107341

[49] Bocanegra-Ibarias P, Garza-González E, Morfín-Otero R, Barrios H, Villarreal-Treviño L, Rodríguez-Noriega E, Garza-Ramos U, Petersen-Morfin S, Silva-Sanchez J (2017) Molecular and microbiological report of a hospital outbreak of NDM-1-carrying enterobacteriaceae in Mexico. PLoS ONE 12:e0179651. 10.1371/journal.pone.0179651 28636666 10.1371/journal.pone.0179651 PMC5479539

[50] Bosch T, Lutgens SPM, Hermans MHA, Wever PC, Schneeberger PM, Renders NHM, Leenders ACAP, Kluytmans JAJW, Schoffelen A, Notermans D, Witteveen S, Bathoorn E, Schouls LM (2017) Outbreak of NDM-1-producing klebsiella pneumoniae in a Dutch hospital, with interspecies transfer of the resistance plasmid and unexpected occurrence in unrelated health care centers. J Clin Microbiol 55:2380–2390. 10.1128/JCM.00535-17 28515215 10.1128/JCM.00535-17 PMC5527415

[51] Witteveen S, Hans JB, Izdebski R, Hasman H, Samuelsen Ø, Dortet L, Pfeifer Y, Delappe N, Oteo-Iglesias J, Żabicka D, Cormican M, Sandfort M, Reichert F, Pöntinen AK, Fischer MA, Verkaik N, Pérez-Vazquez M, Pfennigwerth N, Hammerum AM, Hallstrøm S, Biedrzycka M, Räisänen K, Wielders CC, Urbanowicz P, de Haan A, Westmo K, Landman F, van der Heide HG, Lansu S, Zwittink RD, Notermans DW, Guzek A, Kondratiuk V, Salmanov A, Haller S, Linkevicius M, Gatermann S, Kohlenberg A, Gniadkowski M, Werner G, Hendrickx AP (2024) Dissemination of extensively drug-resistant NDM-producing *Providencia stuartii* in Europe linked to patients transferred from Ukraine, March 2022 to March 2023. Euro Surveill 29:2300616. 10.2807/1560-7917.ES.2024.29.23.2300616 38847120 10.2807/1560-7917.ES.2024.29.23.2300616 PMC11158010

[52] Fasciana T, Antonelli A, Bianco G, Lombardo D, Codda G, Roscetto E, Perez M, Lipari D, Arrigo I, Galia E, Tricoli MR, Calvo M, Niccolai C, Morecchiato F, Errico G, Stefani S, Cavallo R, Marchese A, Catania MR, Ambretti S, Rossolini GM, Pantosti A, Palamara AT, Sabbatucci M, Serra N, Giammanco A (2023) Multicenter study on the prevalence of colonization due to carbapenem-resistant *Enterobacterales* strains before and during the first year of COVID-19, Italy 2018–2020. Front Public Health 11:1270924. 10.3389/fpubh.2023.1270924 38186699 10.3389/fpubh.2023.1270924 PMC10771343

[53] Sader HS, Mendes RE, Carvalhaes CG, Kimbrough JH, Castanheira M (2023) Changing epidemiology of carbapenemases among carbapenem-resistant enterobacterales from United States hospitals and the activity of aztreonam-avibactam against contemporary enterobacterales (2019–2021). Open Forum Infect Dis 10:ofad046. 10.1093/ofid/ofad046 36846612 10.1093/ofid/ofad046 PMC9945928

[54] Thomas GR, Corso A, Pasterán F, Shal J, Sosa A, Pillonetto M, de Souza Peral RT, Hormazábal JC, Araya P, Saavedra SY, Ovalle MV, Jiménez Pearson MA, Chacón GC, Carbon E, Mazariegos Herrera CJ, Velásquez SDCG, Satan-Salazar C, Villavicencio F, Touchet NM, Busignani S, Mayta-Barrios M, Ramírez-Illescas J, Vega ML, Mogdasy C, Rosas V, Salgado N, Quiroz R, El-Omeiri N, Galas MF, Ramón-Pardo P, Melano RG (2022) Increased detection of carbapenemase-producing enterobacterales bacteria in latin America and the caribbean during the COVID-19 pandemic. Emerg Infect Dis 28:1–8. 10.3201/eid2811.220415 36286547 10.3201/eid2811.220415 PMC9622262

[55] van Duin D, Doi Y (2017) The global epidemiology of carbapenemase-producing Enterobacteriaceae. Virulence 8:460–469. 10.1080/21505594.2016.1222343 27593176 10.1080/21505594.2016.1222343 PMC5477705

[56] Sader HS, Carvalhaes CG, Kimbrough JH, Mendes RE, Castanheira M (2024) Activity of aztreonam-avibactam against Enterobacterales resistant to recently approved beta-lactamase inhibitor combinations collected in Europe, Latin America, and the Asia-Pacific Region (2020–2022). Int J Antimicrob Agents 63:107113. 10.1016/j.ijantimicag.2024.107113 38354826 10.1016/j.ijantimicag.2024.107113

[57] Castanheira M, Deshpande LM, Mendes RE, Doyle TB, Sader HS (2022) Prevalence of carbapenemase genes among carbapenem-nonsusceptible *Enterobacterales* collected in US hospitals in a five-year period and activity of ceftazidime/avibactam and comparator agents. JAC Antimicrob Resist 4:dlac098. 10.1093/jacamr/dlac098 36196444 10.1093/jacamr/dlac098 PMC9524567

[58] Rossolini GM, Arhin FF, Kantecki M (2024) In vitro activity of aztreonam-avibactam and comparators against Metallo-β-Lactamase-producing Enterobacterales from ATLAS Global Surveillance Program, 2016–2020. J Glob Antimicrob Resist 36:123–131. 10.1016/j.jgar.2023.12.027 38154750 10.1016/j.jgar.2023.12.027

[59] Bianco G, Boattini M, Cricca M, Diella L, Gatti M, Rossi L, Bartoletti M, Sambri V, Signoretto C, Fonnesu R, Comini S, Gaibani P (2024) Updates on the activity, efficacy and emerging mechanisms of resistance to cefiderocol. Curr Issues Mol Biol 14(46):14132–14133. 10.3390/cimb46120846 10.3390/cimb46120846 PMC11674395 39727974

[60] Karakonstantis S, Rousaki M, Vassilopoulou L, Kritsotakis EI (2024) Global prevalence of cefiderocol non-susceptibility in Enterobacterales, Pseudomonas aeruginosa, Acinetobacter baumannii, and Stenotrophomonas maltophilia: a systematic review and meta-analysis. Clin Microbiol Infect 30:178–188. 10.1016/j.cmi.2023.08.029 37666449 10.1016/j.cmi.2023.08.029

[61] Zhao J, Pu D, Li Z, Liu X, Zhang Y, Wu Y et al (2023) In vitro activity of cefiderocol, a siderophore cephalosporin, against carbapenem-resistant hypervirulent Klebsiella pneumoniae in China. Antimicrob Agents Chemother 67:e0073523. 10.1128/aac.00735-23 38014944 10.1128/aac.00735-23 PMC10720542

[62] Gill CM, Santini D, Nicolau DP (2024) In vitro activity of cefiderocol against a global collection of carbapenem-resistant Pseudomonas aeruginosa with a high level of carbapenemase diversity. J Antimicrob Chemother 79:412–416. 10.1093/jac/dkad396 38153232 10.1093/jac/dkad396 PMC10832583

[63] Findlay J, Raro OHF, Poirel L, Nordmann P (2024) Molecular analysis of metallo-beta-lactamase-producing Pseudomonas aeruginosa in Switzerland 2022–2023. Eur J Clin Microbiol Infect Dis 43:551–557. 10.1007/s10096-024-04752-8 38233610 10.1007/s10096-024-04752-8 PMC10917820

[64] Kayama S, Kawakami S, Kondo K, Kitamura N, Yu L, Hayashi W et al (2024) In vitro activity of cefiderocol against carbapenemase-producing and meropenem-non-susceptible Gram-negative bacteria collected in the Japan antimicrobial resistant bacterial surveillance. J Glob Antimicrob Resist 38:12–20. 10.1016/j.jgar.2024.05.009 38789082 10.1016/j.jgar.2024.05.009

[65] Bianco G, Boattini M, Comini S, Iannaccone M, Casale R, Allizond V et al (2022) Activity of ceftolozane-tazobactam, ceftazidime-avibactam, meropenem-vaborbactam, cefiderocol and comparators against Gram-negative organisms causing bloodstream infections in Northern Italy (2019–2021): emergence of complex resistance phenotypes. J Chemother 34:302–310. 10.1080/1120009X.2022.2031471 35098907 10.1080/1120009X.2022.2031471

[66] Dahdouh E, Gómez-Marcos L, Cañada-García JE, de Arellano ER, Sánchez-García A, Sánchez-Romero I et al (2024) Characterizing carbapenemase-producing *Escherichia coli* isolates from Spain: high genetic heterogeneity and wide geographical spread. Front Cell Infect Microbiol 14:1390966. 10.3389/fcimb.2024.1390966 38817448 10.3389/fcimb.2024.1390966 PMC11137265

[67] Takemura M, Wise MG, Hackel MA, Sahm DF, Yamano Y (2023) In vitro activity of cefiderocol against MBL-producing Gram-negative bacteria collected in North America and Europe in five consecutive annual multinational SIDERO-WT surveillance studies (2014–2019). J Antimicrob Chemother 78:2019–2027. 10.1093/jac/dkad200 37390312 10.1093/jac/dkad200 PMC10393876

[68] Delgado-Valverde M, Portillo-Calderón I, Recacha E, Pérez-Palacios P, Pascual A (2023) *In Vitro* activity of cefiderocol compared to other antimicrobials against a collection of metallo-beta-lactamase-producing gram-negative Bacilli from Southern Spain. Microbiol Spectr 11:e0493622. 10.1128/spectrum.04936-22 37249425 10.1128/spectrum.04936-22 PMC10269457

[69] Huang YS, Chen PY, Chou PC, Wang JT (2023) *In Vitro* activities and inoculum effects of cefiderocol and aztreonam-avibactam against metallo-β-lactamase-producing *Enterobacteriaceae*. Microbiol Spectr 11:e0056923. 10.1128/spectrum.00569-23 37154758 10.1128/spectrum.00569-23 PMC10269523

[70] Buyukyanbolu E, Genc L, Cyr EA, Karakus M, Comert F, Otlu B, Aktas E, Nicolau DP (2024) Antimicrobial susceptibility profile of ceftolozane/tazobactam, ceftazidime/avibactam and cefiderocol against carbapenem-resistant Pseudomonas aeruginosa clinical isolates from Türkiye. Eur J Clin Microbiol Infect Dis 43:1787–1794. 10.1007/s10096-024-04896-7 38995343 10.1007/s10096-024-04896-7

[71] Santerre Henriksen A, Arena F, Attwood M, Canton R, Gatermann S, Naas T, Morrissey I, Longshaw C; ARTEMIS Study Investigators (2024) *In vitro* activity of cefiderocol against European Enterobacterales, including isolates resistant to meropenem and recentβ-lactam/β-lactamase inhibitor combinations. Microbiol Spectr 12: e0418123. 10.1128/spectrum.04181-23 10.1128/spectrum.04181-23 PMC11302063 38904361

[72] Słabisz N, Leśnik P, Janc J, Fidut M, Bartoszewicz M, Dudek-Wicher R, Nawrot U (2024) Evaluation of the *in vitro* susceptibility of clinical isolates of NDM-producing *Klebsiella pneumoniae* to new antibiotics included in a treatment regimen for infections. Front Microbiol 15:1331628. 10.3389/fmicb.2024 38646622 10.3389/fmicb.2024.1331628 PMC11027895

[73] Méndez-Sotelo BJ, Delgado-Beltrán M, Hernández-Durán M, Colín-Castro CA, Esquivel-Bautista J, Ortega-Oliva SA, Ortiz-Álvarez J, García-Contreras R, Franco-Cendejas R, Lopez Jacome LE (2024) In vitro activity of ceftazidime/avibactam, cefiderocol, meropenem/vaborbactam and imipenem/relebactam against clinical strains of the Stenotrophomonas maltophilia complex. PLoS ONE 19:e0298577. 10.1371/journal.pone.0298577 38635685 10.1371/journal.pone.0298577 PMC11025899

[74] Santerre Henriksen A, Jeannot K, Oliver A, Perry JD, Pletz MW, Stefani S, Morrissey I, Longshaw C; ARTEMIS Study Investigators (2024)* In vitro* activity of cefiderocol against European *Pseudomonas aeruginosa* and *Acinetobacter* spp., including isolates resistant to meropenem and recent β-lactam/β-lactamase inhibitor combinations. Microbiol Spectr 12: e0383623. 10.1128/spectrum.03836-23 10.1128/spectrum.03836-23 PMC10986614 38483164

[75] Tunney MM, Elborn JS, McLaughlin CS, Longshaw CM (2024) In vitro activity of cefiderocol against Gram-negative pathogens isolated from people with cystic fibrosis and bronchiectasis. J Glob Antimicrob Resist 36:407–410. 10.1016/j.jgar.2024.01.023 38336228 10.1016/j.jgar.2024.01.023

[76] Huang YS, Chuang YC, Chen PY, Chou PC, Wang JT (2024) *In vitro* activity of cefiderocol and comparator antibiotics against multidrug-resistant non-fermenting Gram-negative bacilli. JAC Antimicrob Resist 6:dlae006. 10.1093/jacamr/dlae006 38304722 10.1093/jacamr/dlae006 PMC10833645

[77] Boattini M, Comini S, Bianco G, Iannaccone M, Casale R, Cavallo R, Costa C (2023) Activity of cefiderocol and synergy of novel β-lactam-β-lactamase inhibitor-based combinations against metallo-β-lactamase-producing gram-negative bacilli: insights from a two-year study (2019–2020). J Chemother 35:198–204. 10.1080/1120009X.2022.2090615 35731718 10.1080/1120009X.2022.2090615

[78] Bassetti M, Echols R, Matsunaga Y, Ariyasu M, Doi Y, Ferrer R, Lodise TP, Naas T, Niki Y, Paterson DL et al (2021) Efficacy and safety of cefiderocol or best available therapy for the treatment of serious infections caused by carbapenem-resistant Gram-negative bacteria (CREDIBLE-CR): a randomised, open-label, multicentre, pathogen-focused, descriptive, phase 3 trial. Lancet Infect Dis 21:226–240. 10.1016/S1473-3099(20)30796-9 33058795 10.1016/S1473-3099(20)30796-9

[79] Wunderink RG, Matsunaga Y, Ariyasu M, Clevenbergh P, Echols R, Kaye KS, Kollef M, Menon A, Pogue JM, Shorr AF et al (2021) Cefiderocol versus high-dose, extended-infusion meropenem for the treatment of Gram-negative nosocomial pneumonia (APEKS-NP): a randomised, double-blind, phase 3, non-inferiority trial. Lancet Infect Dis 21:213–225. 10.1016/S1473-3099(20)30731-3 33058798 10.1016/S1473-3099(20)30731-3

[80] Timsit JF, Paul M, Shields RK, Echols R, Baba T, Yamano Y, Portsmouth S (2022) Cefiderocol for the treatment of infections due to metallo-B-lactamase-producing pathogens in the CREDIBLE-CR and APEKS-NP phase 3 randomized studies. Clin Infect Dis 75:1081–1084. 10.1093/cid/ciac078 35148378 10.1093/cid/ciac078 PMC9522395

[81] Gomis-Font MA, Clari MA, López-Causapé C, Navarro D, Oliver A (2024) Emergence of cefiderocol resistance during ceftazidime/avibactam treatment caused by a large genomic deletion, including *ampD* and *piuCD* genes, in *Pseudomonas aeruginosa*. Antimicrob Agents Chemother 68:e0119223. 10.1128/aac.01192-23 38063398 10.1128/aac.01192-23 PMC10777826

[82] Streling AP, Al Obaidi MM, Lainhart WD, Zangeneh T, Khan A, Dinh AQ, Hanson B, Arias CA, Miller WR (2021) Evolution of cefiderocol non-susceptibility in pseudomonas aeruginosa in a patient without previous exposure to the antibiotic. Clin Infect Dis 73:e4472–e4474. 10.1093/cid/ciaa1909 33411899 10.1093/cid/ciaa1909 PMC8825772

[83] Findlay J, Bianco G, Boattini M, Nordmann P (2024) In vivo development of cefiderocol resistance in carbapenem-resistant Acinetobacter baumannii associated with the downregulation of a TonB-dependent siderophore receptor, PiuA. J Antimicrob Chemother 79:928–930. 10.1093/jac/dkae018 38297993 10.1093/jac/dkae018 PMC10984935

[84] Huang E, Thompson RN, Moon SH, Keck JM, Lowry MS, Melero J, Jun S-R, Rosenbaum ER, Dare RK (2024) Treatment-emergent cefiderocol resistance in carbapenem-resistant *Acinetobacter baumannii* is associated with insertion sequence IS*Aba36* in the siderophore receptor *pirA*. Antimicrob Agents Chemother 68:e0029024. 10.1128/aac.00290-24 38809000 10.1128/aac.00290-24 PMC11232405

[85] Simner PJ, Mostafa HH, Bergman Y, Ante M, Tekle T, Adebayo A, Beisken S, Dzintars K, Tamma PD (2022) progressive development of cefiderocol resistance in escherichia coli during therapy is associated with an increase in blaNDM-5 copy number and gene expression. Clin Infect Dis 75:47–54. 10.1093/cid/ciab888 34618008 10.1093/cid/ciab888 PMC9402677

[86] Bianco G, Boattini M, Comini S, Gibellini D, Gaibani P (2024) In Vivo emergence of ceftazidime/avibactam, cefiderocol and aztreonam/avibactam cross-resistance in a patient with KPC-producing Klebsiella pneumoniae infection after cefiderocol-based treatment. Int J Antimicrob Agents 64:107343. 10.1016/j.ijantimicag.2024.107343 39362613 10.1016/j.ijantimicag.2024.107343

[87] EMA (2024) New antibiotic to fght infections caused by multidrug-resistant bacteria. https://wwwema.europa.eu/en/news/new-antibiotic-fght-infec%20tions-caused-multidrug-resistant-bacteria. Accessed 25 Oct 2024

[88] Paul M, Carrara E, Retamar P et al (2022) European society of clinical microbiology and infectious diseases (ESCMID) guidelines for the treatment of infections caused by multidrug-resistant Gramnegative bacilli (endorsed by European Society of Intensive Care Medicine). Clin Microbiol Infect 28:521–547 34923128 10.1016/j.cmi.2021.11.025

[89] Tamma PD, Aitken SL, Bonomo RA et al (2023) Infectious diseases society of America 2023 guidance on the treatment of antimicrobial resistant gramnegative infections. Clin Infect Dis. 10.1093/cid/ciad428 37463564 10.1093/cid/ciad428

[90] Vázquez-Ucha JC, Alonso-Garcia I, Guijarro-Sánchez P, Lasarte-Monterrubio C, Álvarez-Fraga L, Cendón-Esteve A, Outeda M, Maceiras R, Peña-Escolano A, Martínez-Guitián M, Arca-Suárez J, Bou G, Beceiro A; GEMARA-SEIMC/REIPI Enterobacterales Study Group (2023) Activity of aztreonam in combination with novel β-lactamase inhibitors against metallo-β-lactamase-producing Enterobacterales from Spain. Int J Antimicrob Agents 61: 106738. 10.1016/j.ijantimicag.2023.106738 10.1016/j.ijantimicag.2023.106738 36736925

[91] Chen J, Liu Y, Jia W, Xu X, Sun G, Wang T, Li J, Zhang G, Jing R, Sun H, Xu Y, Liu Y (2023) *In Vitro* activities of aztreonam-avibactam, eravacycline, cefoselis, and other comparators against clinical *Enterobacterales* isolates: a multicenter study in China, 2019. Microbiol Spectr 11:e0487322. 10.1128/spectrum.04873-22 37184411 10.1128/spectrum.04873-22 PMC10269566

[92] Livermore DM, Mushtaq S, Vickers A, Woodford N (2023) Activity of aztreonam/avibactam against metallo-β-lactamase-producing Enterobacterales from the UK: impact of penicillin-binding protein-3 inserts and CMY-42 β-lactamase in Escherichia coli. Int J Antimicrob Agents 61:106776. 10.1016/j.ijantimicag.2023.106776 36893810 10.1016/j.ijantimicag.2023.106776

[93] Sader HS, Mendes RE, Arends SJR, Carvalhaes CG, Castanheira M (2022) Antimicrobial activities of aztreonam-avibactam and comparator agents tested against Enterobacterales from European hospitals analysed by geographic region and infection type (2019–2020). Eur J Clin Microbiol Infect Dis 41:477–487. 10.1007/s10096-022-04400-z 35041100 10.1007/s10096-022-04400-z

[94] Esposito S, Stone GG, Papaparaskevas J (2021) In vitro activity of aztreonam/avibactam against a global collection of Klebsiella pneumoniae collected from defined culture sources in 2016 and 2017. J Glob Antimicrob Resist 24:14–22. 10.1016/j.jgar.2020.08.004 32841721 10.1016/j.jgar.2020.08.004

[95] Zhang B, Zhu Z, Jia W, Qu F, Huang B, Shan B, Yu H, Tang Y, Chen L, Du H (2020) In vitro activity of aztreonam-avibactam against metallo-β-lactamase-producing Enterobacteriaceae-a multicenter study in China. Int J Infect Dis 97:11–18. 10.1016/j.ijid.2020.05.075.7 32473388 10.1016/j.ijid.2020.05.075

[96] Karlowsky JA, Kazmierczak KM, de Jonge BLM, Hackel MA, Sahm DF, Bradford PA (2017) *In Vitro* activity of aztreonam-avibactam against enterobacteriaceae and pseudomonas aeruginosa isolated by clinical laboratories in 40 countries from 2012 to 2015. Antimicrob Agents Chemother 61:e00472-e517. 10.1128/AAC.00472-17 28630192 10.1128/AAC.00472-17 PMC5571336

[97] Biedenbach DJ, Kazmierczak K, Bouchillon SK, Sahm DF, Bradford PA (2015) In vitro activity of aztreonam-avibactam against a global collection of Gram-negative pathogens from 2012 and 2013. Antimicrob Agents Chemother 59:4239–4248. 10.1128/AAC.00206-15 25963984 10.1128/AAC.00206-15 PMC4468705

[98] Blanco-Martín T, López-Hernández I, Aracil B, González-Pinto L, Aja-Macaya P, Alonso-García I, Rodríguez-Pallares S, Sánchez-Peña L, Outeda-García M, Pérez-Vázquez M, Vázquez-Ucha JC, Beceiro A, Pascual Á, Bou G, López-Cerero L, Oteo-Iglesias J, Arca-Suárez J; GEMARA-SEIMC/CIBERINFEC Study Group on the activity and resistance mechanisms to new β-lactams and β-lactamase inhibitors (PROTECT) (2024) Assessment of the activity and mechanisms of resistance to cefiderocol and combinations of β-lactams and the novel β-lactamase inhibitors avibactam, taniborbactam, zidebactam, nacubactam, xeruborbactam, and ANT3310 in emerging double-carbapenemase-producing Enterobacterales. Antimicrob Agents Chemother 68: e0092424. 10.1128/aac.00924-24 10.1128/aac.00924-24 PMC11539232 39382274

[99] Mauri C, Maraolo AE, Di Bella S, Luzzaro F, Principe L (2021) The revival of aztreonam in combination with avibactam against metallo-β-lactamase-producing gram-negatives: a systematic review of in vitro studies and clinical cases. Antibiotics (Basel) 10:1012. 10.3390/antibiotics10081012 34439062 10.3390/antibiotics10081012 PMC8388901

[100] Biagi M, Lamm D, Meyer K, Vialichka A, Jurkovic M, Patel S, Mendes RE, Bulman ZP, Wenzler E (2020) Activity of aztreonam in combination with avibactam, clavulanate, relebactam, and vaborbactam against multidrug-resistant stenotrophomonas maltophilia. Antimicrob Agents Chemother 64(12):10–1128. 10.1128/AAC.00297-20 10.1128/AAC.00297-20 PMC7674038 32928733

[101] Sader HS, Duncan LR, Arends SJR, Carvalhaes CG, Castanheira M (2020) Antimicrobial activity of aztreonam-avibactam and comparator agents when tested against a large collection of contemporary stenotrophomonas maltophilia isolates from medical centers worldwide. Antimicrob Agents Chemother 64:e01433-e1520. 10.1128/AAC.01433-20 32900683 10.1128/AAC.01433-20 PMC7577171

[102] Cornely OA, Cisneros JM, Torre-Cisneros J et al (2020) Pharmacokinetics and safety of aztreonam/avibactam for the treatment of complicated intra-abdom- inal infections in hospitalized adults: results from the REJUVENATE study. J Antimicrob Chemother 75:618–627 31828337 10.1093/jac/dkz497 PMC7021089

[103] Lodise TP, Smith NM, O’Donnell N et al (2020) Determining the optimal dosing of a novel combination regimen of ceftazidime/avibactam with aztreonam against NDM-1-producing Enterobacteriaceae using a hollow-fbre infection model. J Antimicrob Chemother 75:2622–2632 32464664 10.1093/jac/dkaa197 PMC8444334

[104] Carmeli Y, Cisneros JM, Paul M, Daikos GL, Wang M, Cisneros JT, Singer G, Titov I, Gumenchuk I, Zhao Y, Jiménez Rodríguez RM, Liang L, Chen G, Pyptiuk O, Aksoy F, Rogers H, Wible M, Arhin F, Luckey A, Leaney J, Pypstra R, Chow J (2023) 2893 A. Efficacy and safety of aztreonam-avibactam for the treatment of serious infections due to gram-negative bacteria, including metallo-β-lactamase-producing pathogens: phase 3 REVISIT study. Open Forum Infect Dis 10:ofad500.2476. 10.1093/ofid/ofad500.2476

[105] Simner PJ, Bergman Y, Conzemius R et al (2023) An NDM-producing *Escherichia coli* clinical isolate exhibiting resistance to cefiderocol and the combination of ceftazidime-avibactam and aztreonam: another step toward pan-β-lactam resistance. Open Forum Infect Dis 10:ofad276. 10.1093/ofid/ofad276 37416757 10.1093/ofid/ofad276 PMC10319620

[106] Haidar G, Kline EG, Kitsios GD, Wang X, Kwak EJ, Newbrough A, Friday K, Hughes Kramer K, Shields RK (2024) Emergence of high-level aztreonam-avibactam and cefiderocol resistance following treatment of an NDM-producing *Escherichia coli* bloodstream isolate exhibiting reduced susceptibility to both agents at baseline. JAC Antimicrob Resist 6:dlae141. 10.1093/jacamr/dlae141 39239090 10.1093/jacamr/dlae141 PMC11375572

[107] Periasamy H, Joshi P, Palwe S, Shrivastava R, Bhagwat S, Patel M (2020) High prevalence of *Escherichia coli* clinical isolates in India harbouring four amino acid inserts in PBP3 adversely impacting activity of aztreonam/avibactam. J Antimicrob Chemother 75:1650–1651 32040179 10.1093/jac/dkaa021

[108] Wang Q, Jin L, Sun S et al (2022) Occurrence of high levels of cefiderocol resistance in carbapenem-resistant *Escherichia coli* before its approval in China: a report from China CRE-network. Microbiol Spectr 10:e0267021 35481835 10.1128/spectrum.02670-21 PMC9241927

[109] Rossolini GM, Stone G, Kantecki M, Arhin FF (2022) In vitro activity of aztreonam/avibactam against isolates of Enterobacterales collected globally from ATLAS in 2019. J Glob Antimicrob Resist 30:214–221 35760303 10.1016/j.jgar.2022.06.018

[110] Poirel L, de la Rosa JMO, Sakaoglu Z, Kusaksizoglu A, Sadek M, Nordmann P (2022) NDM-35-Producing ST167 *Escherichia coli* highly resistant to beta-lactams including cefiderocol. Antimicrob Agents Chemother 66:e0031122 35867524 10.1128/aac.00311-22 PMC9380521

[111] Martin MJ, Luo TL, Kovalchuk V, Kondratiuk V, Dao HD, Kovalenko I, Plaza BJ, Kettlewell JM, Anderson CP, Smedberg JR, Ong AC, Kwak YI, Hawley-Molloy JS, Bennett JW, McGann PT, Lebreton F (2024) Detection of cefiderocol and aztreonam/avibactam resistance in epidemic *Escherichia coli* ST-361 carrying *bla*_NDM-5_ and *bla*_KPC-3_ from foreign fighters evacuated from Ukraine. Antimicrob Agents Chemother 20:e0109024. 10.1128/aac.01090-24 10.1128/aac.01090-24 PMC11539215 39302119

[112] Sadek M, Juhas M, Poirel L et al (2020) Genetic features leading to reduced susceptibility to aztreonam-avibactam among metallo-β-lactamase-producing *Escherichia coli* isolates. Antimicrob Agents Chemother 64:e01659-e1720. 10.1128/AAC.01659-20 32988825 10.1128/AAC.01659-20 PMC7674043

[113] Findlay J, Poirel L, Kessler J et al (2021) New Delhi metallo-β-lactamase-producing enterobacterales bacteria, Switzerland, 2019–2020. Emerg Infect Dis 27:2628–2637. 10.3201/eid2710.211265 34545787 10.3201/eid2710.211265 PMC8462332

[114] Alm RA, Johnstone MR, Lahiri SD (2015) Characterization of *Escherichia coli* NDM isolates with decreased susceptibility to aztreonam/avibactam: role of a novel insertion in PBP3. J Antimicrob Chemother 70:1420–1428. 10.1093/jac/dku568 25634992 10.1093/jac/dku568

[115] Sato T, Ito A, Ishioka Y, Matsumoto S, Rokushima M, Kazmierczak KM, Hackel M, Sahm DF, Yamano Y (2020) *Escherichia coli* strains possessing a four amino acid YRIN insertion in PBP3 identified as part of the SIDERO-WT-2014 surveillance study. JAC Antimicrob Resist 2:dlaa081. 10.1093/jacamr/dlaa081 34223033 10.1093/jacamr/dlaa081 PMC8210206

[116] Zhang Y, Kashikar A, Brown CA et al (2017) Unusual *Escherichia coli* PBP 3 insertion sequence identified from a collection of carbapenem-resistant Enterobacteriaceae tested in vitro with a combination of ceftazidime-, ceftaroline-, or aztreonam-avibactam. Antimicrob Agents Chemother 61:e00389-e417. 10.1128/AAC.00389-17 28559260 10.1128/AAC.00389-17 PMC5527577

[117] Nordmann P, Yao Y, Falgenhauer L et al (2021) Recent emergence of aztreonam-avibactam resistance in NDM and OXA-48 carbapenemase-producing *Escherichia coli* in Germany. Antimicrob Agents Chemother 65:e0109021. 10.1128/AAC.01090-21 34424048 10.1128/AAC.01090-21 PMC8522741

[118] Di Pilato V, Codda G, Niccolai C, Willison E, Wong JLC, Coppo E, Frankel G, Marchese A, Rossolini GM (2024) Functional features of KPC-109, a novel 270-loop KPC-3 mutant mediating resistance to avibactam-based β-lactamase inhibitor combinations and cefiderocol. Int J Antimicrob Agents 63:107030. 10.1016/j.ijantimicag.2023.107030 37931849 10.1016/j.ijantimicag.2023.107030

[119] Lang PA, Parkova A, Leissing TM, Calvopiña K, Cain R, Krajnc A, Panduwawala TD, Philippe J, Fishwick CWG, Trapencieris P, Page MGP, Schofield CJ, Brem J (2020) Bicyclic boronates as potent inhibitors of AmpC, the class C β-lactamase from *Escherichia coli*. Biomolecules 10:899. 10.3390/biom10060899 32545682 10.3390/biom10060899 PMC7356297

[120] Liu B, Trout REL, Chu GH, McGarry D, Jackson RW, Hamrick JC, Daigle DM, Cusick SM, Pozzi C, De Luca F, Benvenuti M, Mangani S, Docquier JD, Weiss WJ, Pevear DC, Xerri L, Burns CJ (2020) Discovery of taniborbactam (VNRX-5133): a broad-spectrum serine- and metallo-β-lactamase inhibitor for carbapenem-resistant bacterial infections. J Med Chem 63(6):2789–2801. 10.1021/acs.jmedchem.9b01518 31765155 10.1021/acs.jmedchem.9b01518 PMC7104248

[121] Piccirilli A, Segatore B, Brisdelli F, Amicosante G, Perilli M (2021) Potent inhibitory activity of taniborbactam towards NDM-1 and NDM-1^Q119X^ mutants, and in vitro activity of cefepime/taniborbactam against MBLs producing Enterobacterales. Int J Antimicrob Agents 57:106228. 10.1016/j.ijantimicag.2020.106228 33246038 10.1016/j.ijantimicag.2020.106228

[122] Asempa TE, Kuti JL, Nascimento JC, Pope SJ, Salerno EL, Troy PJ, Nicolau DP (2023) Bronchopulmonary disposition of IV cefepime/taniborbactam (2–0.5 g) administered over 2 h in healthy adult subjects. J Antimicrob Chemother 78:703–709. 10.1093/jac/dkac447 36617636 10.1093/jac/dkac447 PMC9978582

[123] Hamrick JC, Docquier JD, Uehara T, Myers CL, Six DA, Chatwin CL, John KJ, Vernacchio SF, Cusick SM, Trout REL, Pozzi C, De Luca F, Benvenuti M, Mangani S, Liu B, Jackson RW, Moeck G, Xerri L, Burns CJ, Pevear DC, Daigle DM (2020) VNRX-5133 (Taniborbactam), a broad-spectrum inhibitor of serine- and metallo-β-lactamases, restores activity of cefepime in *Enterobacterales* and pseudomonas aeruginosa. Antimicrob Agents Chemother 64:e01963-e2019. 10.1128/AAC.01963-19 31871094 10.1128/AAC.01963-19 PMC7038240

[124] Karlowsky JA, Hackel MA, Wise MG, Six DA, Uehara T, Daigle DM, Cusick SM, Pevear DC, Moeck G, Sahm DF (2023) *In Vitro* activity of cefepime-taniborbactam and comparators against clinical isolates of gram-negative bacilli from 2018 to 2020: results from the global evaluation of antimicrobial resistance via surveillance (GEARS) program. Antimicrob Agents Chemother 67:e0128122. 10.1128/aac.01281-22 36541767 10.1128/aac.01281-22 PMC9872668

[125] Wang X, Zhao C, Wang Q, Wang Z, Liang X, Zhang F, Zhang Y, Meng H, Chen H, Li S, Zhou C, Li H, Wang H (2020) In vitro activity of the novel β-lactamase inhibitor taniborbactam (VNRX-5133), in combination with cefepime or meropenem, against MDR Gram-negative bacterial isolates from China. J Antimicrob Chemother 75:1850–1858. 10.1093/jac/dkaa053. (**Erratum.In:JAntimicrobChemother.2020(75),pp.10,2019.1093/jac/dkaa132**) 32154866 10.1093/jac/dkaa053

[126] Mushtaq S, Vickers A, Doumith M, Ellington MJ, Woodford N, Livermore DM (2021) Activity of β-lactam/taniborbactam (VNRX-5133) combinations against carbapenem-resistant Gram-negative bacteria. J Antimicrob Chemother 76:160–170. 10.1093/jac/dkaa391 33305800 10.1093/jac/dkaa391

[127] Meletiadis J, Paranos P, Georgiou PC, Vourli S, Antonopoulou S, Michelaki A, Vagiakou E, Pournaras S (2021) In vitro comparative activity of the new beta-lactamase inhibitor taniborbactam with cefepime or meropenem against Klebsiella pneumoniae and cefepime against Pseudomonas aeruginosa metallo-beta-lactamase-producing clinical isolates. Int J Antimicrob Agents 58:106440. 10.1016/j.ijantimicag.2021.106440 34551356 10.1016/j.ijantimicag.2021.106440

[128] Vázquez-Ucha JC, Lasarte-Monterrubio C, Guijarro-Sánchez P, Oviaño M, Álvarez-Fraga L, Alonso-García I, Arca-Suárez J, Bou G, Beceiro A; GEMARA-SEIMC/REIPI Enterobacterales Study Group (2022) Assessment of activity and resistance mechanisms to cefepime in combination with the novel β-lactamase inhibitors zidebactam, taniborbactam, and enmetazobactam against a multicenter collection of carbapenemase-producing *Enterobacterales*. Antimicrob Agents Chemother 66:e0167621. 10.1128/AAC.01676-21 10.1128/AAC.01676-21 PMC8846464 34807754

[129] Hernández-García M, García-Castillo M, Ruiz-Garbajosa P, Bou G, Siller-Ruiz M, Pitart C, Gracia-Ahufinger I, Mulet X, Pascual Á, Tormo N, Cantón R (2022) *In Vitro* activity of cefepime-taniborbactam against carbapenemase-producing *Enterobacterales* and pseudomonas aeruginosa isolates recovered in Spain. Antimicrob Agents Chemother 66:e0216121. 10.1128/aac.02161-21 35007130 10.1128/aac.02161-21 PMC8923209

[130] Bakthavatchalam YD, Elangovan D, Jaganathan SV, Subburaju N, Shankar A, Manokaran Y, J S, Devi R, Baveja S, Devi S, S J, Bhattacharya S, S M R, Yesudhason B, Shetty V, Mutreja A, Manesh A, Varghese GM, Marwick CA, Parcell BJ, Gilbert IH, Veeraraghavan B (2023) *In Vitro* activity of two cefepime-based novel combinations, cefepime/taniborbactam and cefepime/zidebactam, against carbapenemase-expressing *Enterobacterales* collected in India. Microbiol Spectr 11: e0492522. 10.1128/spectrum.04925-22 10.1128/spectrum.04925-22 PMC10100882 36847537

[131] Golden AR, Baxter MR, Karlowsky JA, Mataseje L, Mulvey MR, Walkty A, Bay D, Schweizer F, Lagace-Wiens PRS, Adam HJ, Zhanel GG (2022) Activity of cefepime/taniborbactam and comparators against whole genome sequenced ertapenem-non-susceptible Enterobacterales clinical isolates: CANWARD 2007–19. JAC Antimicrob Resist 4:dlab197. 10.1093/jacamr/dlab197 35156028 10.1093/jacamr/dlab197 PMC8826793

[132] Ono D, Mojica MF, Bethel CR, Ishii Y, Drusin SI, Moreno DM, Vila AJ, Bonomo RA (2024) Structural role of K224 in taniborbactam inhibition of NDM-1. Antimicrob Agents Chemother 68(2):e0133223. 10.1128/aac.01332-23 38174924 10.1128/aac.01332-23 PMC10848753

[133] Drusin SI, Le Terrier C, Poirel L, Bonomo RA, Vila AJ, Moreno DM (2024) Structural basis of metallo-β-lactamase resistance to taniborbactam. Antimicrob Agents Chemother 68:e0116823. 10.1128/aac.01168-23 38063400 10.1128/aac.01168-23 PMC10848773

[134] Le Terrier C, Nordmann P, Buchs C, Di DYW, Rossolini GM, Stephan R, Castanheira M, Poirel L (2023) Wide dissemination of Gram-negative bacteria producing the taniborbactam-resistant NDM-9 variant: a One Health concern. J Antimicrob Chemother 78:2382–2384. 10.1093/jac/dkad210 37394537 10.1093/jac/dkad210 PMC10477121

[135] Le Terrier C, Viguier C, Nordmann P, Vila AJ, Poirel L (2024) Relative inhibitory activities of the broad-spectrum β-lactamase inhibitor taniborbactam against metallo-β-lactamases. Antimicrob Agents Chemother 68:e0099123. 10.1128/aac.00991-23 38047644 10.1128/aac.00991-23 PMC10848752

[136] Drusin SI, Le Terrier C, Poirel L, Bonomo RA, Vila AJ, Moreno DM (2024) Structural basis of metallo-β-lactamase resistance to taniborbactam. Antimicrob Agents Chemother 68(2):e0116823. 10.1128/aac.01168-23 38063400 10.1128/aac.01168-23 PMC10848773

[137] Wagenlehner FM, Gasink LB, McGovern PC, Moeck G, McLeroth P, Dorr M, Dane A, Henkel T; CERTAIN-1 Study Team (2024) Cefepime-taniborbactam in complicated urinary tract infection. N Engl J Med 390:611–622. 10.1056/NEJMoa2304748 10.1056/NEJMoa2304748 38354140

[138] Hecker SJ, Reddy KR, Lomovskaya O, Griffith DC, Rubio-Aparicio D, Nelson K, Tsivkovski R, Sun D, Sabet M, Tarazi Z, Parkinson J, Totrov M, Boyer SH, Glinka TW, Pemberton OA, Chen Y, Dudley MN (2020) Discovery of cyclic boronic acid QPX7728, an ultrabroad-spectrum inhibitor of serine and metallo-β-lactamases. J Med Chem 63:7491–7507. 10.1021/acs.jmedchem.9b01976 32150407 10.1021/acs.jmedchem.9b01976

[139] Tsivkovski R, Totrov M, Lomovskaya O (2020) Biochemical characterization of QPX7728, a new ultrabroad-spectrum beta-lactamase inhibitor of serine and metallo-beta-lactamases. Antimicrob Agents Chemother 64:e00130-e220. 10.1128/AAC.00130-20 32152086 10.1128/AAC.00130-20 PMC7269513

[140] Lomovskaya O, Tsivkovski R, Nelson K, Rubio-Aparicio D, Sun D, Totrov M, Dudley MN (2020) Spectrum of Beta-lactamase inhibition by the cyclic boronate QPX7728, an ultrabroad-spectrum beta-lactamase inhibitor of serine and metallo-beta-lactamases: enhancement of activity of multiple antibiotics against isogenic strains expressing single beta-lactamases. Antimicrob Agents Chemother 64:e00212-e220. 10.1128/AAC.00212-20 32229489 10.1128/AAC.00212-20 PMC7269471

[141] Le Terrier C, Freire S, Viguier C, Findlay J, Nordmann P, Poirel L (2024) Relative inhibitory activities of the broad-spectrum β-lactamase inhibitor xeruborbactam in comparison with taniborbactam against metallo-β-lactamases produced in *Escherichia coli* and *Pseudomonas aeruginosa*. Antimicrob Agents Chemother e0157023. 10.1128/aac.01570-23 10.1128/aac.01570-23 PMC11620488 38727224

[142] Nelson K, Rubio-Aparicio D, Sun D, Dudley M, Lomovskaya O (2020) *In Vitro* activity of the ultrabroad-spectrum-beta-lactamase inhibitor QPX7728 against carbapenem-resistant *Enterobacterales* with varying intrinsic and acquired resistance mechanisms. Antimicrob Agents Chemother 64:e00757-e820. 10.1128/AAC.00757-20 32482673 10.1128/AAC.00757-20 PMC7526838

[143] Lomovskaya O, Castanheira M, Lindley J, Rubio-Aparicio D, Nelson K, Tsivkovski R, Sun D, Totrov M, Loutit J, Dudley M (2023) *In vitro* potency of xeruborbactam in combination with multiple β-lactam antibiotics in comparison with other β-lactam/β-lactamase inhibitor (BLI) combinations against carbapenem-resistant and extended-spectrum β-lactamase-producing *Enterobacterales*. Antimicrob Agents Chemother 67:e0044023. 10.1128/aac.00440-23 37800963 10.1128/aac.00440-23 PMC10648875

[144] Nelson K, Rubio-Aparicio D, Tsivkovski R, Sun D, Totrov M, Dudley M, Lomovskaya O (2020) *In Vitro* activity of the ultra-broad-spectrum beta-lactamase inhibitor QPX7728 in combination with meropenem against clinical isolates of carbapenem-resistant acinetobacter baumannii. Antimicrob Agents Chemother 64(11):e01406-e1420. 10.1128/AAC.01406-20 32868334 10.1128/AAC.01406-20 PMC7577151

[145] Lomovskaya O, Rubio-Aparicio D, Nelson K, Sun D, Tsivkovski R, Castanheira M, Lindley J, Loutit J, Dudley M (2021) *In Vitro* Activity of the ultrabroad-spectrum beta-lactamase inhibitor QPX7728 in combination with multiple beta-lactam antibiotics against pseudomonas aeruginosa. Antimicrob Agents Chemother 65:e00210-e221. 10.1128/AAC.00210-21 33782010 10.1128/AAC.00210-21 PMC8315991

[146] Lomovskaya O, Rubio-Aparicio D, Tsivkovski R, Loutit J, Dudley M (2022) The ultrabroad-spectrum beta-lactamase inhibitor QPX7728 restores the potency of multiple oral beta-lactam antibiotics against beta-lactamase-producing strains of resistant *Enterobacterales*. Antimicrob Agents Chemother 66:e0216821. 10.1128/AAC.02168-21 34902261 10.1128/aac.02168-21 PMC8846479

[147] Blanco-Martín T, Alonso-García I, González-Pinto L, Outeda-García M, Guijarro-Sánchez P, López-Hernández I, Pérez-Vázquez M, Aracil B, López-Cerero L, Fraile-Ribot P, Oliver A, Vázquez-Ucha JC, Beceiro A, Bou G, Arca-Suárez J; GEMARA/SEIMC-CIBERINFEC Study Group on the activity and resistance mechanisms to new β-lactams and β-lactamase inhibitors (PROTECT) (2024) Activity of cefiderocol and innovative β-lactam/β-lactamase inhibitor combinations against isogenic strains of Escherichia coli expressing single and double β-lactamases under high and low permeability conditions. Int J Antimicrob Agents 63:107150. 10.1016/j.ijantimicag.2024.107150 Erratum in: Int J Antimicrob Agents. 2024; 64:107264. 10.1016/j.ijantimicag.2024.107264

[148] Papp-Wallace KM, Nguyen NQ, Jacobs MR, Bethel CR, Barnes MD, Kumar V, Bajaksouzian S, Rudin SD, Rather PN, Bhavsar S, Ravikumar T, Deshpande PK, Patil V, Yeole R, Bhagwat SS, Patel MV, van den Akker F, Bonomo RA (2018) Strategic approaches to overcome resistance against gram-negative pathogens using β-lactamase inhibitors and β-lactam enhancers: activity of three novel diazabicyclooctanes WCK 5153, zidebactam (WCK 5107), and WCK 4234. J Med Chem 61:4067–4086. 10.1021/acs.jmedchem.8b00091 29627985 10.1021/acs.jmedchem.8b00091 PMC6131718

[149] Livermore DM, Mushtaq S, Warner M, Vickers A, Woodford N (2017) In vitro activity of cefepime/zidebactam (WCK 5222) against Gram-negative bacteria. J Antimicrob Chemother 72:1373–1385. 10.1093/jac/dkw593 28158732 10.1093/jac/dkw593

[150] Karlowsky JA, Hackel MA, Bouchillon SK, Sahm DF (2020) *In Vitro* activity of WCK 5222 (Cefepime-Zidebactam) against worldwide collected gram-negative bacilli not susceptible to carbapenems. Antimicrob Agents Chemother 64:e01432-e1520. 10.1128/AAC.01432-20 32928739 10.1128/AAC.01432-20 PMC7674044

[151] Kuo SC, Wang YC, Tan MC, Huang WC, Shiau YR, Wang HY, Lai JF, Huang IW, Lauderdale TL (2021) In vitro activity of imipenem/relebactam, meropenem/vaborbactam, ceftazidime/avibactam, cefepime/zidebactam and other novel antibiotics against imipenem-non-susceptible Gram-negative bacilli from Taiwan. J Antimicrob Chemother 76:2071–2078. 10.1093/jac/dkab141 33956969 10.1093/jac/dkab141 PMC8561265

[152] Mushtaq S, Garello P, Vickers A, Woodford N, Livermore DM (2021) Activity of cefepime/zidebactam (WCK 5222) against “problem” antibiotic-resistant Gram-negative bacteria sent to a national reference laboratory. J Antimicrob Chemother 76:1511–1522. 10.1093/jac/dkab067 33760082 10.1093/jac/dkab067

[153] Bhagwat SS, Legakis NJ, Skalidis T, Loannidis A, Goumenopoulos C, Joshi PR, Shrivastava R, Palwe SR, Periasamy H, Patel MV, Chatzipanagiotou S; Hellenic Cefepime/Zidebactam Study Group (2021) In vitro activity of cefepime/zidebactam (WCK 5222) against recent Gram-negative isolates collected from high resistance settings of Greek hospitals. Diagn Microbiol Infect Dis 100: 115327. 10.1016/j.diagmicrobio.2021.115327 10.1016/j.diagmicrobio.2021.115327 33744624

[154] Guo Y, Han R, Jiang B, Ding L, Yang F, Zheng B, Yang Y, Wu S, Yin D, Zhu D, Hu F; China Antimicrobial Surveillance Network (CHINET) Study Group (2022) *In Vitro* activity of new β-lactam-β-lactamase inhibitor combinations and comparators against clinical isolates of gram-negative bacilli: results from the China antimicrobial surveillance network (CHINET) in 2019. Microbiol Spectr 10: e0185422. 10.1128/spectrum.01854-22 10.1128/spectrum.01854-22 PMC9431184 35862963

[155] Sader HS, Mendes RE, Duncan LR, Carvalhaes CG, Castanheria M (2022) Antimicrobial activity of cefepime/zidebactam (WCK 5222), a β-lactam/β-lactam enhancer combination, against clinical isolates of Gram-negative bacteria collected worldwide (2018–19). J Antimicrob Chemother 77:2642–2649. 10.1093/jac/dkac233 35897129 10.1093/jac/dkac233

[156] Hujer AM, Marshall SH, Mack AR, Hujer KM, Bakthavatchalam YD, Umarkar K, Palwe SR, Takalkar S, Joshi PR, Shrivastava R, Periasamy H, Bhagwat SS, Patel MV, Veeraraghavan B, Bonomo RA (2023) Transcending the challenge of evolving resistance mechanisms in *Pseudomonas aeruginosa* through β-lactam-enhancer-mechanism-based cefepime/zidebactam. mBio 14:e0111823. 10.1128/mbio.01118-23 10.1128/mbio.01118-23 PMC10746216 37889005

[157] Lasarte-Monterrubio C, Fraile-Ribot PA, Vázquez-Ucha JC, Cabot G, Guijarro-Sánchez P, Alonso-García I, Rumbo-Feal S, Galán-Sánchez F, Beceiro A, Arca-Suárez J, Oliver A, Bou G (2022) Activity of cefiderocol, imipenem/relebactam, cefepime/taniborbactam and cefepime/zidebactam against ceftolozane/tazobactam- and ceftazidime/avibactam-resistant Pseudomonas aeruginosa. J Antimicrob Chemother 77:2809–2815. 10.1093/jac/dkac241 35904000 10.1093/jac/dkac241

[158] Le Terrier C, Nordmann P, Sadek M, Poirel L (2023) In vitro activity of cefepime/zidebactam and cefepime/taniborbactam against aztreonam/avibactam-resistant NDM-like-producing Escherichia coli clinical isolates. J Antimicrob Chemother 78:1191–1194. 10.1093/jac/dkad061 36921067 10.1093/jac/dkad061 PMC10154122

[159] Pan X, Zhao X, Song Y, Ren H, Tian Z, Liang Q, Jin Y, Bai F, Cheng Z, Feng J, Wu W (2022) Molecular Characterization of WCK 5222 (Cefepime/Zidebactam)-resistant mutants developed from a carbapenem-resistant pseudomonas aeruginosa clinical isolate. Microbiol Spectr 10:e0267821. 10.1128/spectrum.02678-21 35196805 10.1128/spectrum.02678-21 PMC8865557

[160] Rajavel M, Kumar V, Nguyen H, Wyatt J, Marshall SH, Papp-Wallace KM, Deshpande P, Bhavsar S, Yeole R, Bhagwat S, Patel M, Bonomo RA, van den Akker F (2021) Structural characterization of diazabicyclooctane β-lactam "Enhancers" in complex with penicillin-binding proteins PBP2 and PBP3 of Pseudomonas aeruginosa. mBio 12: e03058–20. 10.1128/mBio.03058-20 10.1128/mBio.03058-20 PMC8545096 33593978

[161] González-Pinto L, Alonso-García I, Blanco-Martín T, Camacho-Zamora P, Fraile-Ribot PA, Outeda-García M, Lasarte-Monterrubio C, Guijarro-Sánchez P, Maceiras R, Moya B, Juan C, Vázquez-Ucha JC, Beceiro A, Oliver A, Bou G, Arca-Suárez J (2024) Impact of chromosomally encoded resistance mechanisms and transferable β-lactamases on the activity of cefiderocol and innovative β-lactam/β-lactamase inhibitor combinations against Pseudomonas aeruginosa. J Antimicrob Chemother 79:2591–2597. 10.1093/jac/dkae263 39073766 10.1093/jac/dkae263 PMC11441999

[162] Joshi P, Shrivastava R, Bhagwat S, Patel M (2021) Activity of β-lactam plus β-lactam-enhancer combination cefepime/zidebactam against Klebsiella pneumoniae harbouring defective OmpK35/36 porins and carbapenemases. Diagn Microbiol Infect Dis 101:115481. 10.1016/j.diagmicrobio.2021.115481 34332307 10.1016/j.diagmicrobio.2021.115481

[163] Barceló I, Cabot G, Palwe S, Joshi P, Takalkar S, Periasamy H, Cortés-Lara S, Zamorano L, Sánchez-Diener I, Moya B, Bhagwat S, Patel M, Oliver A (2021) In vitro evolution of cefepime/zidebactam (WCK 5222) resistance in Pseudomonas aeruginosa: dynamics, mechanisms, fitness trade-off and impact on in vivo efficacy. J Antimicrob Chemother 76:2546–2557. 10.1093/jac/dkab213 34219168 10.1093/jac/dkab213

[164] Moya B, Bhagwat S, Cabot G, Bou G, Patel M, Oliver A (2020) Effective inhibition of PBPs by cefepime and zidebactam in the presence of VIM-1 drives potent bactericidal activity against MBL-expressing *Pseudomonas aeruginosa*. J Antimicrob Chemother 75:1474–1478. 10.1093/jac/dkaa036 32083659 10.1093/jac/dkaa036

[165] Moya B, Barcelo IM, Bhagwat S, Patel M, Bou G, Papp-Wallace KM, Bonomo RA, Oliver A (2017) WCK 5107 (Zidebactam) and WCK 5153 are novel inhibitors of PBP2 showing potent “β-lactam enhancer” activity against *Pseudomonas aeruginosa*, including multidrug-resistant metallo-β-lactamase-producing high-risk clones. Antimicrob Agents Chemother 61:e02529-e2616. 10.1128/AAC.02529-16 28289035 10.1128/AAC.02529-16 PMC5444176

[166] Wang L, Zhang X, Zhou X, Bi Y, Wang M, Guo Q, Yang F (2023) Insertion of IS*Pa1635* in IS*CR1* creates a hybrid promoter for *bla*_PER-1_ resulting in resistance to novel β-lactam/β-lactamase inhibitor combinations and cefiderocol. Antimicrob Agents Chemother 67:e0013523. 10.1128/aac.00135-23 37212660 10.1128/aac.00135-23 PMC10269150

[167] Monogue ML, Tabor-Rennie J, Abdelraouf K, Nicolau DP (2019) *In Vivo* efficacy of WCK 5222 (Cefepime-Zidebactam) against multidrug-resistant *Pseudomonas aeruginosa* in the neutropenic murine thigh infection model. Antimicrob Agents Chemother 63:e00233-e319. 10.1128/AAC.00233-19 31235557 10.1128/AAC.00233-19 PMC6591597

[168] Kidd JM, Abdelraouf K, Nicolau DP (2020) Efficacy of human-simulated bronchopulmonary exposures of cefepime, zidebactam and the combination (WCK 5222) against MDR Pseudomonas aeruginosa in a neutropenic murine pneumonia model. J Antimicrob Chemother 75:149–155. 10.1093/jac/dkz414 31641765 10.1093/jac/dkz414

[169] Lasko MJ, Abdelraouf K, Nicolau DP (2021) Comparative in vivo activity of human-simulated plasma and epithelial lining fluid exposures of WCK 5222 (cefepime/zidebactam) against KPC- and OXA-48-like-producing Klebsiella pneumoniae in the neutropenic murine pneumonia model. J Antimicrob Chemother 76:2310–2316. 10.1093/jac/dkab183 34096601 10.1093/jac/dkab183

[170] Tirlangi PK, Wanve BS, Dubbudu RR, Yadav BS, Kumar LS, Gupta A, Sree RA, Challa HPR, Reddy PN (2023) Successful use of cefepime-zidebactam (WCK 5222) as a salvage therapy for the treatment of disseminated extensively drug-resistant New Delhi Metallo-β-lactamase-producing pseudomonas aeruginosa infection in an adult patient with acute T-cell leukemia. Antimicrob Agents Chemother 67:e0050023. 10.1128/aac.00500-23 37314343 10.1128/aac.00500-23 PMC10433839

[171] Dubey D, Roy M, Shah TH, Bano N, Kulshrestha V, Mitra S, Sangwan P, Dubey M, Imran A, Jain B, Velmurugan A, Bakthavatchalam YD, Veeraraghavan B (2023) Compassionate use of a novel β-lactam enhancer-based investigational antibiotic cefepime/zidebactam (WCK 5222) for the treatment of extensively-drug-resistant NDM-expressing Pseudomonas aeruginosa infection in an intra-abdominal infection-induced sepsis patient: a case report. Ann Clin Microbiol Antimicrob 22:55. 10.1186/s12941-023-00606-x 37408075 10.1186/s12941-023-00606-x PMC10324185

[172] Soman R, Sirsat R, Sunavala A, Punatar N, Mehta J, Rodrigues C, Veeraraghavan B (2024) Successful treatment of sino-pulmonary infection & skull base osteomyelitis caused by New Delhi metallo-β-lactamase-producing Pseudomonas aeruginosa in a renal transplant recipient by using an investigational antibiotic cefepime/zidebactam (WCK 5222). Eur J Clin Microbiol Infect Dis. 10.1007/s10096-024-04791-1 38416290 10.1007/s10096-024-04791-1

[173] Morinaka A, Tsutsumi Y, Yamada M, Suzuki K, Watanabe T, Abe T, Furuuchi T, Inamura S, Sakamaki Y, Mitsuhashi N, Ida T, Livermore DM (2015) OP0595, a new diazabicyclooctane: mode of action as a serine β-lactamase inhibitor, antibiotic and β-lactam “enhancer.” J Antimicrob Chemother 70:2779–2786. 10.1093/jac/dkv166 26089439 10.1093/jac/dkv166

[174] Mushtaq S, Vickers A, Woodford N, Haldimann A, Livermore DM (2019) Activity of nacubactam (RG6080/OP0595) combinations against MBL-producing Enterobacteriaceae. J Antimicrob Chemother 74:953–960. 10.1093/jac/dky522 30590470 10.1093/jac/dky522

[175] Le Terrier C, Nordmann P, Poirel L (2022) In vitro activity of aztreonam in combination with newly developed β-lactamase inhibitors against MDR Enterobacterales and Pseudomonas aeruginosa producing metallo-β-lactamases. J Antimicrob Chemother 78:101–107. 10.1093/jac/dkac360 36308322 10.1093/jac/dkac360

[176] Hagihara M, Kato H, Sugano T, Okade H, Sato N, Shibata Y, Sakanashi D, Asai N, Koizumi Y, Suematsu H, Yamagishi Y, Mikamo H (2021) Pharmacodynamic evaluation of meropenem, cefepime, or aztreonam combined with a novel β-lactamase inhibitor, nacubactam, against carbapenem-resistant and/or carbapenemase-producing Klebsiella pneumoniae and Escherichia coli using a murine thigh-infection model. Int J Antimicrob Agents 57:106330. 10.1016/j.ijantimicag.2021.106330 33789129 10.1016/j.ijantimicag.2021.106330

[177] Igarashi Y, Takemura W, Liu X, Kojima N, Morita T, Chuang VTG, Enoki Y, Taguchi K, Matsumoto K (2023) Development of an optimized and practical pharmacokinetics/pharmacodynamics analysis method for aztreonam/nacubactam against carbapenemase-producing K. pneumoniae. J Antimicrob Chemother 78:991–9. 10.1093/jac/dkad033 36775998 10.1093/jac/dkad033 PMC10068424

[178] Monogue ML, Giovagnoli S, Bissantz C, Zampaloni C, Nicolau DP (2018) *In Vivo* efficacy of meropenem with a novel non-β-lactam-β-lactamase inhibitor, nacubactam, against gram-negative organisms exhibiting various resistance mechanisms in a murine complicated urinary tract infection model. Antimicrob Agents Chemother 62:e02596-e2617. 10.1128/AAC.02596-17 30012751 10.1128/AAC.02596-17 PMC6125527

[179] Mallalieu NL, Winter E, Fettner S, Patel K, Zwanziger E, Attley G, Rodriguez I, Kano A, Salama SM, Bentley D, Geretti AM (2020) Safety and pharmacokinetic characterization of nacubactam, a novel β-lactamase inhibitor, alone and in combination with meropenem, in healthy volunteers. Antimicrob Agents Chemother 64:e02229-e2319. 10.1128/AAC.02229-19 32041717 10.1128/AAC.02229-19 PMC7179653

[180] Keam SJ (2023) Sulbactam/durlobactam: first approval. Drugs 83(13):1245–1252. 10.1007/s40265-023-01920-6 37523122 10.1007/s40265-023-01920-6

[181] Kaye KS, Shorr AF, Wunderink RG, Du B, Poirier GE, Rana K, Miller A, Lewis D, O’Donnell J, Chen L, Reinhart H, Srinivasan S, Isaacs R, Altarac D (2023) Efficacy and safety of sulbactam-durlobactam versus colistin for the treatment of patients with serious infections caused by Acinetobacter baumannii-calcoaceticus complex: a multicentre, randomised, active-controlled, phase 3, non-inferiority clinical trial (ATTACK). Lancet Infect Dis 23(9):1072–1084. 10.1016/S1473-3099(23)00184-6 37182534 10.1016/S1473-3099(23)00184-6

[182] Shapiro AB (2017) Kinetics of sulbactam hydrolysis by β-lactamases, and kinetics of β-lactamase inhibition by sulbactam. Antimicrob Agents Chemother 61(12):e01612-e1617. 10.1128/AAC.01612-17 28971872 10.1128/AAC.01612-17 PMC5700308

[183] Durand-Réville TF, Guler S, Comita-Prevoir J, Chen B, Bifulco N, Huynh H, Lahiri S, Shapiro AB, McLeod SM, Carter NM, Moussa SH, Velez-Vega C, Olivier NB, McLaughlin R, Gao N, Thresher J, Palmer T, Andrews B, Giacobbe RA, Newman JV, Ehmann DE, de Jonge B, O’Donnell J, Mueller JP, Tommasi RA, Miller AA (2017) ETX2514 is a broad-spectrum β-lactamase inhibitor for the treatment of drug-resistant Gram-negative bacteria including Acinetobacter baumannii. Nat Microbiol 2:17104. 10.1038/nmicrobiol.2017.104 28665414 10.1038/nmicrobiol.2017.104

[184] Durand-Réville TF, Guler S, Comita-Prevoir J, Chen B, Bifulco N, Huynh H, Lahiri S, Shapiro AB, McLeod SM, Carter NM, Moussa SH, Velez-Vega C, Olivier NB, McLaughlin R, Gao N, Thresher J, Palmer T, Andrews B, Giacobbe RA, Newman JV, Ehmann DE, de Jonge B, O’Donnell J, Mueller JP, Tommasi RA, Miller AA (2017) ETX2514 is a broad-spectrum β-lactamase inhibitor for the treatment of drug-resistant Gram-negative bacteria including Acinetobacter baumannii. Nat Microbiol 30(2):17104. 10.1038/nmicrobiol.2017.104 10.1038/nmicrobiol.2017.104 28665414

[185] McLeod SM, Moussa SH, Hackel MA, Miller AA (2020) In Vitro activity of sulbactam-durlobactam against acinetobacter baumannii-calcoaceticus complex isolates collected globally in 2016 and 2017. Antimicrob Agents Chemother 64(4):e02534-e2619. 10.1128/AAC.02534-19 31988095 10.1128/AAC.02534-19 PMC7179289

[186] Principe L, Di Bella S, Conti J, Perilli M, Piccirilli A, Mussini C, Decorti G (2022) Acinetobacter baumannii Resistance to sulbactam/durlobactam: a systematic review. Antibiotics (Basel) 11(12):1793. 10.3390/antibiotics11121793 36551450 10.3390/antibiotics11121793 PMC9774100

[187] Aitken SL, Pierce VM, Pogue JM, Kline EG, Tverdek FP, Shields RK (2024) The growing threat of ndm-producing escherichia coli with penicillin-binding protein 3 mutations in the United States-is there a potential role for durlobactam? Clin Infect Dis 79(4):834–837. 10.1093/cid/ciae229 . Erratum.In:ClinInfectDis.2024Sep,20(ciae414),pp.10.1093/cid/ciae414 10.1093/cid/ciae229 PMC12098015 38661186

[188] Le Terrier C, Nordmann P, Delaval A; NARA Network; Poirel L (2024) Potent in-vitro activity of sulbactam-durlobactam against NDM-producing Escherichia coli including cefiderocol and aztreonam-avibactam-resistant isolates. Clin Microbiol Infect S1198–743X(24)00494–4. 10.1016/j.cmi.2024.10.012. 10.1016/j.cmi.2024.10.012 39447746

